# The triacylglycerol, hydroxytriolein, inhibits triple negative mammary breast cancer cell proliferation through a mechanism dependent on dihydroceramide and Akt

**DOI:** 10.18632/oncotarget.26824

**Published:** 2019-04-02

**Authors:** Francisca Guardiola-Serrano, Roberto Beteta-Göbel, Raquel Rodríguez-Lorca, Maitane Ibarguren, David J. López, Silvia Terés, María Alonso-Sande, Mónica Higuera, Manuel Torres, Xavier Busquets, Pablo V. Escribá

**Affiliations:** ^1^ Laboratory of Molecular Cell Biomedicine, Department of Biology, University of the Balearic Islands, E-07122 Palma, Balearic Islands, Spain

**Keywords:** hydroxytriolein, membrane lipid therapy, lipid composition and cancer treatment, autophagy, triple negative breast cancer

## Abstract

The plasma membrane is an attractive target for new anticancer drugs, not least because regulating its lipid structure can control multiple signaling pathways involved in cancer cell proliferation, differentiation and survival. Accordingly, the novel anticancer drug hydroxytriolein (HTO) was designed to interact with and regulate the composition and structure of the membrane, which in turn controls the interaction of amphitropic signaling membrane proteins with the lipid bilayer. Changes in signaling provoked by HTO impair the growth of triple negative breast cancer (TNBC) cells, aggressive breast tumor cells that have a worse prognosis than other types of breast cancers and for which there is as yet no effective targeted therapy. HTO alters the lipid composition and structure of cancer cell membranes, inhibiting the growth of MDA-MB-231 and BT-549 TNBC cells *in vitro*. Depending on the cellular context, HTO could regulate two pathways involved in TNBC cell proliferation. On the one hand, HTO might stimulate ERK signaling and induce TNBC cell autophagy, while on the other, it could increase dihydroceramide and ceramide production, which would inhibit Akt independently of EGFR activation and provoke cell death. *In vivo* studies using a model of human TNBC show that HTO and its fatty acid constituent (2-hydroxyoleic acid) impair tumor growth, with no undesired side effects. For these reasons, HTO appears to be a promising anticancer molecule that targets the lipid bilayer (membrane-lipid therapy). By regulating membrane lipids, HTO controls important signaling pathways involved in cancer cell growth, the basis of its pharmacological efficacy and safety.

## INTRODUCTION

Breast cancer is the most common cancer in women, accounting for 25–29% of all cancers. Moreover, it is the first cause of cancer related death among women in non-developed regions and it ranks second in developed countries [1, 2]. Mortality has been reduced in breast cancer patients by a combination of early diagnosis and targeted therapies. Breast cancer therapy mainly targets the estrogen receptor (ER), the progesterone receptor (PR) or human epidermal growth factor receptor 2 (HER-2). Triple Negative Breast Cancer (TNBC) represents a heterogeneous group of breast cancers [3, 4] that are characterized by the absence of these three receptors and they account for 15–20% of all breast cancers. The loss of these receptors is associated with a higher relapse rate, more aggressiveness and a worse prognosis than that associated with other types of breast cancer [5, 6]. Moreover, there is currently no specific targeted therapy to combat TNBC, and the main therapeutic approach is chemotherapy with taxanes, platinum salts and anthracyclines [7]. Nevertheless, TNBC has a worse disease free survival and overall survival rate than other types of breast cancer.

Membrane-lipid therapy (MLT) is an innovative therapeutic approach to treat cancer that targets the composition and structure of cell membranes. Thus, MLT based compounds control tumor growth [8–10] by modifying the biophysical properties of the lipid membrane, which in turn affects the activity of relevant signaling proteins and their downstream pathways [11–15]. This regulation of membrane lipids can influence the raft-to-non-raft microdomain balance, which in turn controls the distribution of signaling proteins and hence, their interactions with membrane receptors and effectors involved in cancer cell growth [13].

A high fat diet is an important modulator of breast cancer risk [16, 17], with dietary energy more relevant than dietary fat in tumorigenesis. In fact, the type of fat in the diet is an important risk factor for cancer and for example, the Mediterranean diet is associated with a reduced incidence of cancer [18]. Olive oil is one of the main sources of fat in the Mediterranean diet and it has been the focus of several studies on cancer. Some epidemiological studies have shown that olive oil consumption reduces the incidence of breast cancer [19–22] and olive oil not only reduces breast cancer incidence in animal models but also, its aggressiveness. Indeed, olive oil consumption dampens Akt activation in human tumors [23]. Triolein (TO) represents ca. 50% of the triacylglycerol species in olive oil, and its oleic acid (OA) fatty acyl moiety is the main lipid in this food (70–80%). OA has antitumor effects against some types of breast cancer cell lines, employing molecular mechanisms that vary depending on the type of cancer cell. Thus, OA potentiates the effects of aromatase inhibitors via glutathione depletion in ER positive tumors [24], while it synergistically enhances the growth inhibitory effect of trastuzumab in HER-2 positive breast cells, repressing HER-2 transcription [25, 26].

The epidermal growth factor receptor (EGFR) is a molecular target currently under study to treat TNBC [7, 27]. More than 50% of TNBCs strongly express EGFR and these cancers respond poorly to chemotherapy [28]. Recent reports show resistance to receptor tyrosine kinase (RTK) inhibitors is correlated to EGFR lipid raft localization in breast cancer cells, and that disrupting lipid rafts by cholesterol depletion sensitizes these resistant breast cancer cells to EGFR inhibitors, dampening Akt activation [29]. In this context, both OA and its derivative, 2OHOA, enhance plasma membrane fluidity, and they destabilize liquid ordered (lipid raft) domains in the lipid bilayer [15].

We recently showed that a more stable TO analogue, hydroxytriolein (HTO), has a stronger antitumor efficacy than TO against non-small cell lung cancer (NSCLC). HTO is a 2-hydroxy fatty-acyl TO derivative that regulates membrane lipid structure, favoring the membrane translocation and activation of PKC and ERK, as well as the production of reactive oxygen species (ROS) and macroautophagy specifically in cancer but not in normal cells [30]. Here, we have used different human TNBC cell lines to evaluate the antitumor potential of HTO. While HTO did not modify the amount of EGFR in lipid raft membrane microdomains, it did enhance ERK signaling and dihydroceramide production, with a concomitant increase in cancer cell death. Here, we used MDA-MB-231, Hs-578T, BT-549 cells as models of TNBC cells. Although these cell lines share their epithelial nature and mammary gland origin, they showed some differences in their response to HTO, which could be due to the heterogeneity of the molecular alterations involved in their diverted tumorigenic processes. The present study opens new avenues for the treatment of this currently resistant breast cancers.

## RESULTS

### HTO impairs TNBC cell proliferation and viability

**Figure 1:**
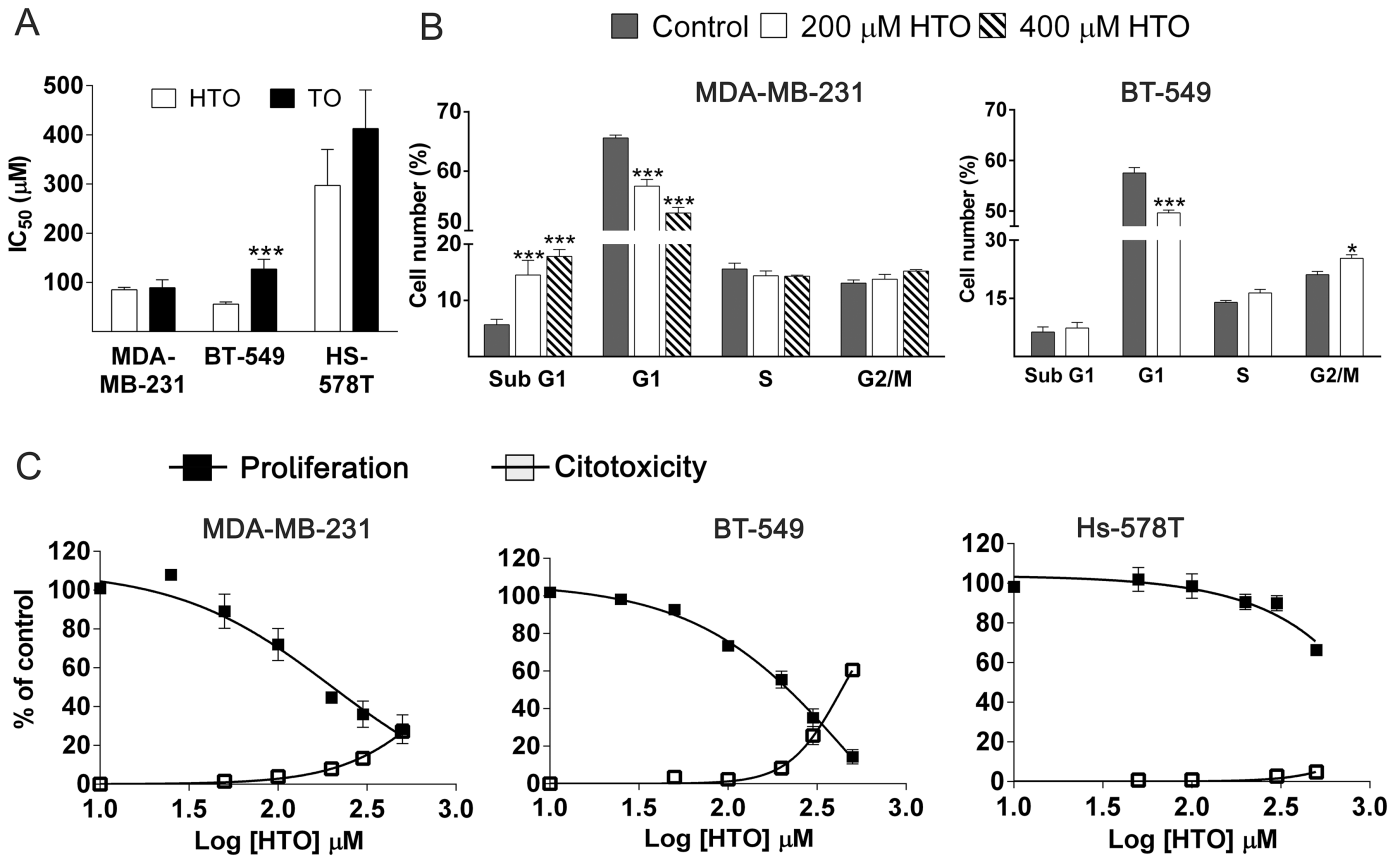
Efficacy of HTO and TO against TNBC cell proliferation (**A**) IC_50_ values for HTO and TO against TNBC cell proliferation after 48 h treatments (mean ± SEM from 3 independent experiments performed in triplicate). (**B**) Cells were cultured in the presence or absence of HTO for 24 h or 48 h, fixed with ethanol, treated with RNase and stained with propidium iodide. The cell cycle distribution was analyzed and represented as the percentage of the total cell number (mean ± SEM of 2 independent experiments performed in triplicate). (**C**) Concentration-dependent cell growth inhibition (mean ± SEM of 3 independent experiments) and induction of cytotoxicity (determined by LDH release as indicated in the Materials and Methods section) in MDA-MB-231, BT-549 and Hs-578T TNBC cells (mean ± SEM of 2 independent experiments performed in triplicate): ^*^p < 0.05, ^**^p < 0.01, ^***^p < 0.001.

Exposing TNBC cells to HTO or TO (prepared as described previously [30]) reduced the number of viable cells in culture in a dose- and time-dependent manner. After 48 h, HTO more strongly affected BT-549 and Hs-5748T cells than TO (IC_50_ 59.2 ± 4.4 μM and 297 ± 73 μM for HTO; 146.6 ± 5 μM vs 412.5 ± 78 μM for TO, respectively: Figure 1A). To determine whether the effect of HTO on cell proliferation was a result of cell cycle arrest or cell death, we analyzed the cell cycle distribution of a population of MDA-MB-231 cells by flow cytometry using propidium iodide. Exposing these cells to HTO (200–400 μM) for 24–48 h reduced the population in the G_1_ phase of cell growth, with a concomitant increase in the population of cells in the subG_1_ phase, these cells undergoing cell death (Figure 1B). When BT-549 cells were treated with HTO (200 μM) for 48 h, the population of cells in the G_2_-M phase augmented, with a concomitant decrease of the cells in the G_0_/G_1_ phase, suggesting the induction of cell cycle arrest (Figure 1B). The SubG_1_ peak in cytometry profiles may increase as a consequence of either apoptosis or necrosis, as cells or cell fragments with reduced DNA content can be produced by different cell death types. Plasma membrane permeation serves as a hallmark of necrotic cell death and it can be measured by the release of lactate dehydrogenase (LDH). Indeed, after 24 h in the presence of HTO (300 μM) we detected LDH release in 13% of MDA-MB-231 and 26% of BT-549 cells (Figure 1C). Together, these data suggest that exposure to HTO provokes cell death through diverse mechanisms.

### HTO and TO regulate the lipid composition of the membrane

**Figure 2:**
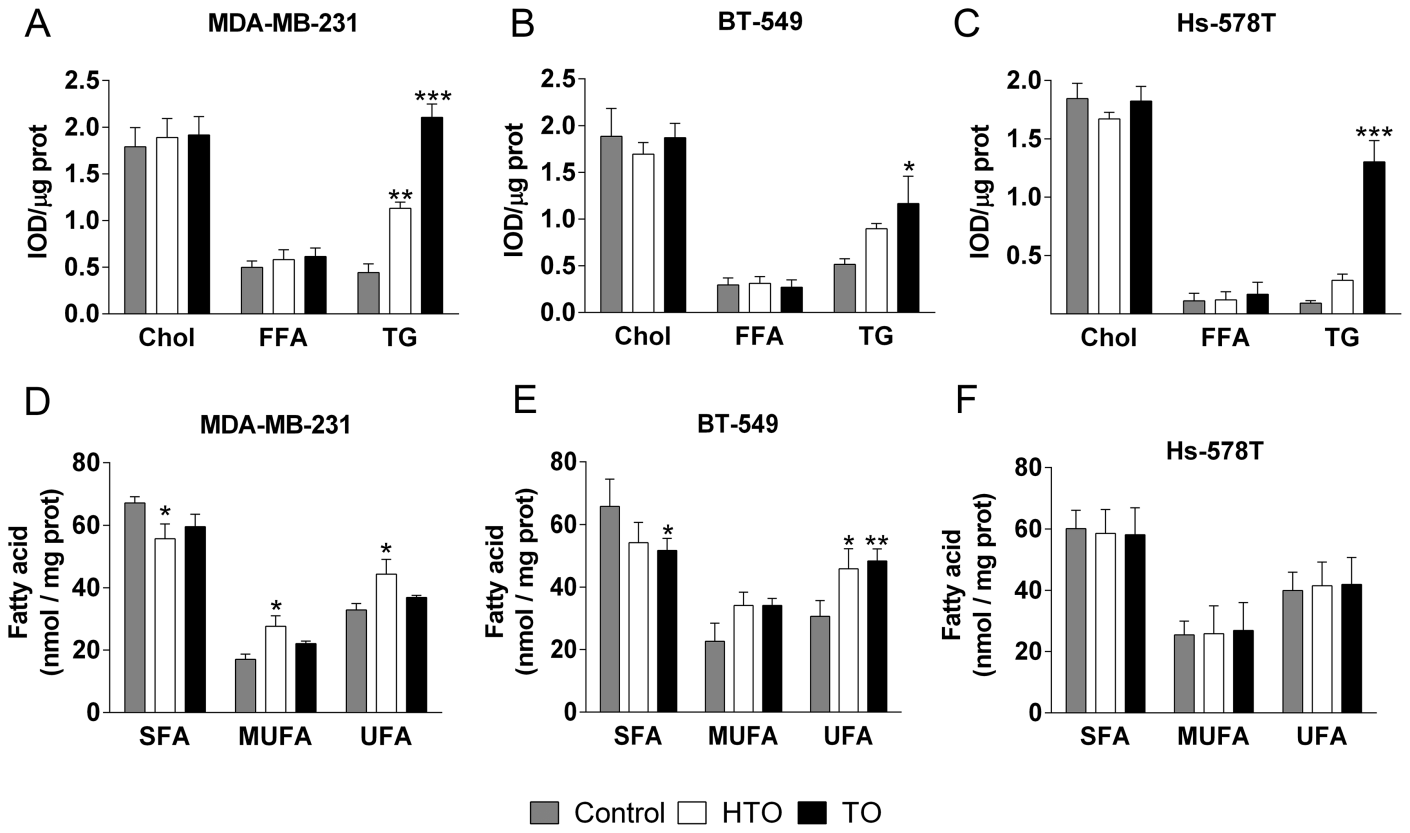
Effect of HTO and TO on cell lipids The MDA-MB-231, BT-549 and Hs-578T TNBC lines were cultured for 24 h in the presence or absence of HTO or TO (300 μM) before their lipids were extracted and fractionated either by TLC to measure neutral lipids (**A**–**C**) or by GC to measure the fatty acid levels (**D**–**F**) Chol, cholesterol; TGs, triacylglycerols; FFA, free fatty acids; SFA, saturated fatty acid; MUFA, monounsaturated fatty acids and UFA, unsaturated fatty acid. The bars correspond to the mean ± SEM from 2 independent experiments: ^*^p < 0.05, ^**^p < 0.01, ^***^p < 0.001 vs control.

**Figure 3:**
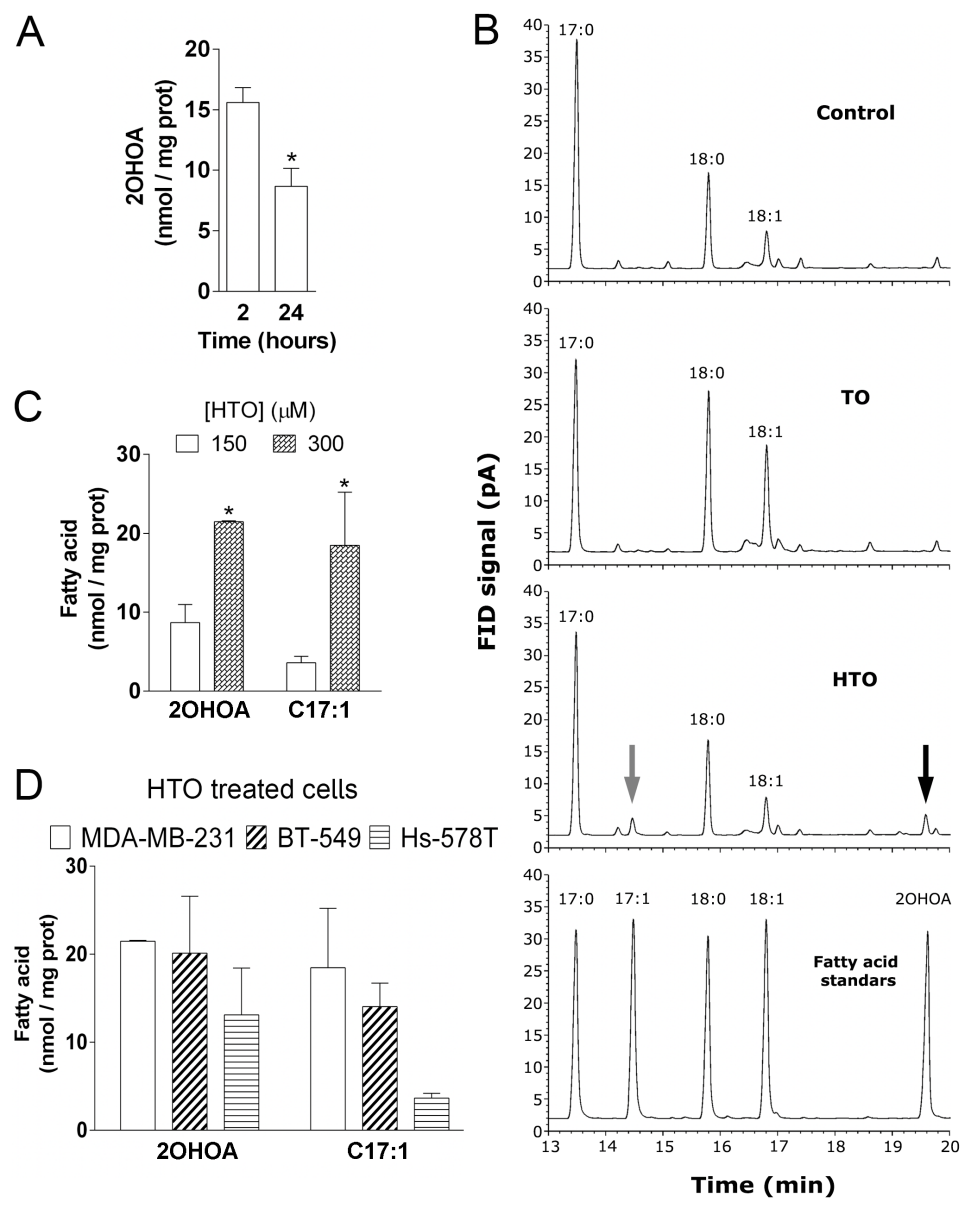
Effect of HTO and TO on membrane fatty acid composition in MDA-MB-231 cells MDA-MB-231 cells were maintained in the presence or absence of HTO (150 μM) for 2 or 24 h before their membranes were isolated and their lipids extracted. Fatty acid levels were quantified by GC and identified by comparison with standards. (**A**) The levels of 2OHOA in HTO treated cells. (**B**) Amplified region of representative chromatograms showing the fatty acid composition in MDA-MB231 cells treated with the vehicle alone (Control, upper panel), TO (second panel) or HTO (third panel). In cells exposed to HTO, the grey arrow indicates the peak corresponding to C17:1 and the black arrow corresponds to 2OHOA, as shown in the bottom panel (fatty acid standards). (**C**) Quantification of 2OHOA and C17:1 in MDA-MB-231 cells cultured in the presence of HTO (150 or 300 μM). (**D**) Quantification of 2OHOA and C17:1 in TNBC cells (MDA-MB-231, BT-549 and Hs-578T) cultured in the presence of 300 μM HTO: ^*^p < 0.05.

Changes in the lipid composition of the membrane can induce distinct events, such as cancer cell proliferation or quiescence. We analyzed the effects of HTO and TO on the membrane lipid composition of TNBC cells by liquid and gas chromatography (LC and GC). Thin layer chromatography (HP-TLC) showed that the triacylglycerol (TG) content increased in all TO-treated cell lines, with the strongest increase in Hs-578T cells (ca. 14-fold with respect to the untreated controls), followed by MDA-MB-231 (5.4-fold) and BT-549 (3.6-fold) cells (Figure 2A–2C). By contrast, HTO only produced significant changes in TG content in MDA-MB231 cells (2.9-fold increase). No differences in the phospholipid composition of the membrane were evident after a 24 h or 48 h exposure to either compound (Supplementary Figure 1). When the fatty acid content of the cells was analyzed by GC, the saturated-to-unsaturated fatty acid ratio increased significantly in MDA-MB-231 cells treated with HTO (0.8 vs 0.5 for untreated cells) and in BT-549 cells treated with TO (0.9 vs 0.5 for untreated cells: Figure 2D–2F), this parameter affecting the biophysical properties of the membrane. In this context, there was a significant increase in palmitic (C16:0), stearic (C18:0) and oleic (C18:1) acids in all TO treated cells, yet not in those exposed to HTO (Table 1). Conversely, cells exposed to HTO displayed 2 fatty acid peaks corresponding to 2OHOA (C18:1) and heptadecenoic acid (HDA, C17:1). 2OHOA is the fatty acid present in HTO and HDA could be produced by the α-oxidation of 2OHOA, and indeed, the concentration of both lipids was directly correlated with the concentration of HTO in cultures (Figure 3). These results indicated that HTO and TO were processed through different metabolic pathways and consequently, that they produced distinct changes in the membrane lipid profile of TNBC cells.

**Table 1:** Fatty acid levels (nmol/mg protein)

MDA-MB-231					
FA species	Control	HTO 150 μM	HTO 300 μM	TO 150 μM	TO 300 μM
**14:0**	8.1 ± 1.2	9.3 ± 0.9	10.7 ± 4.3	9.3 ± 1.1	25.0 ± 10.0
**16:0**	91.5 ± 13.0	103.0 ± 12.5	114.2 ± 31.7	138.8 ± 5.4^***^	170.4 ± 43.7^***^
**16:1n-7**	2.7 ± 0.2	3.7 ± 0.4	5.4 ± 2.6	7.1 ± 0.6	5.7 ± 1.8
**18:0**	133.6 ± 13.0	133.4 ± 12.0	144.0 ± 15.6	261.3 ± 34.5^***^	199.8 ± 36.7^***^
**18:1n-9**	56.9 ± 4.5	62.1 ± 4.7	99.4 ± 45.4^*^	194.6 ± 24.0^***^	126.1 ± 47.9^***^
**18:2n-6**	6.5 ± 1.0	9.5 ± 0.9	10.8 ± 4.3	20.8 ± 5.0	9.8 ± 4.2
**18:3n-3**	0.7 ± 0.3	2.4 ± 1.6	4.9 ± 2.3	0.8 ± 0.8	26.7 ± 6.5
**20:0**	2.5 ± 0.3	3.6 ± 1.1	7.1 ± 4.7	5.4 ± 0.7	3.7 ± 0.5
**20:1n-9**	2.5 ± 1.8	0.5 ± 0.5	24.9 ± 22.6	0.7 ± 0.7	2.1 ± 0.4
**20:4n-6**	22.8 ± 3.9	31.5 ± 4.7	14.2 ± 4.24	70.1 ± 0.7^***^	13.5 ± 4.6
**20:5n-3**	2.3 ± 0.6	5.0 ± 0.5	3.8 ± 1.0	10.3 ± 1.2	3.0 ± 0.5
**22:6n-3**	10.4 ± 1.2	13.6 ± 1.8	9.5 ± 0.4	30.4 ± 5.6	5.1 ± 2.6
**24:1n-9**	2.3 ± 0.6	4.0 ± 0.5	2.8 ± 0.4	8.7 ± 1.4	1.7 ± 1.1
	**BT-549**			**HS-578T**		
**FA species**	**Control**	**HTO 300 μM**	**TO 300 μM**	**Control**	**HTO 300 μM**	**TO 300 μM**
**14:0**	7.3 ± 3.07	6.9 ± 1.3	12.4 ± 2.1	4.3 ± 0.93	5.5 ± 1.0	5.9 ± 1.6
**16:0**	118.5 ± 33.2	107.7 ± 6.4	140.8 ± 2.0	67.2 ± 11.3	83.7 ± 11.5	88.5 ± 15.4^*^
**16:1n-7**	4.8 ± 0.46	6.3 ± 1.0	10.3 ± 0.6	3.0 ± 0.2	3.2 ± 0.4	3.9 ± 0.2
**18:0**	131.5 ± 53.2	114.2 ± 17.6	116.6 ± 12.1	59.2 ± 14.5	78.9 ± 21.6	88.7 ± 24.8^**^
**18:1n-9**	79.9 ± 5.2	102.7 ± 15.5	164.9 ± 7.8^***^	47.1 ± 2.5	48.5 ± 1.8	68.7 ± 4.1^*^
**18:2n-6**	4.1 ± 0.08	6.9 ± 1.4	10.8 ± 0.3	6.4 ± 0.4	6.4 ± 0.4	6.6 ± 0.5
**18:3n-6**	1.6 ± 0.2	3.4 ± 2.0	11.1 ± 4.0	ND	0.3 ± 0.3	ND
**20:0**	2.2 ± 0.74	2.6 ± 0.3	3.2 ± 0.6	1.0 ± 0.1	1.2 ± 0.3	1.8 ± 0.3
**20:1n-9**	2.8 ± 0.4	5.6 ± 1.4	7.5 ± 1.3	1.2 ± 0.2	1.5 ± 0.1	1.2 ± 0.5
**20:4n-6**	16.2 ± 1.4	19.6 ± 1.2	18.4 ± 1.5	16.9 ± 1.8	13.1 ± 3.2	18.9 ± 0.1
**20:5n-3**	3.3 ± 0.4	3.8 ± 0.6	3.9 ± 1.0	2.9 ± 0.1	3.2 ± 0.7	2.7 ± 0.3
**22:6n-3**	6.3 ± 0.4	7.4 ± 0.8	6.4 ± 1.0	5.2 ± 0.2	5.4 ± 0.7	5.2 ± 0.1
**24:1n-9**	7.2 ± 0.4	10.1 ± 0.6	8.2 ± 0.5	3.57 ± 0.2	3.3 ± 1.1	5.3 ± 0.4

The values in the table correspond to the mean ± SEM expressed in nmol lipid/mg protein: ^*^p < 0.05, ^**^p < 0.01, ^***^p < 0.001 vs control. FA, fatty acid.

### HTO modulates cell signaling distinctly in different TNBC cell lines

**Figure 4:**
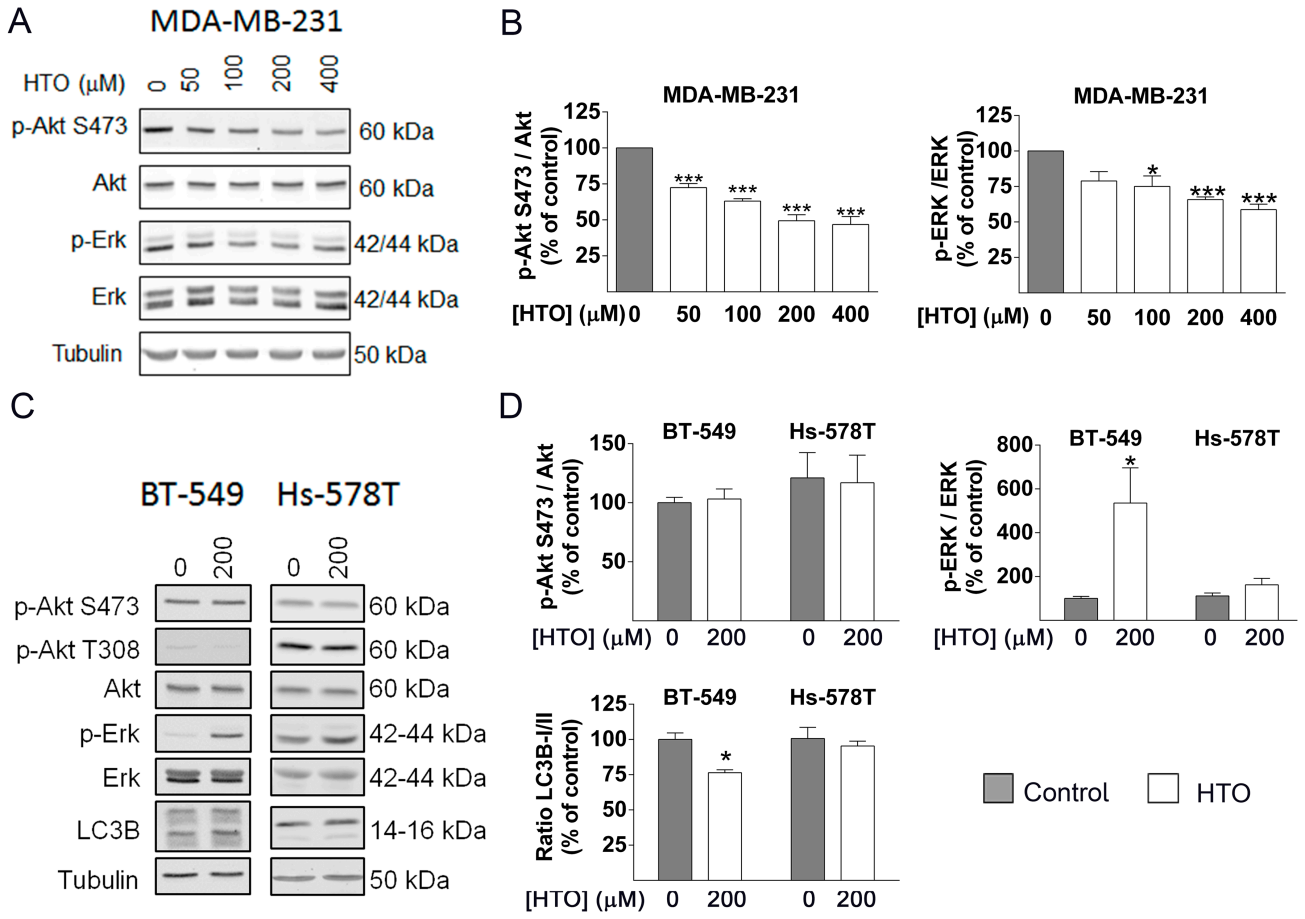
Effect of HTO on cell signaling MDA-MB-231 (**A**–**B**), BT-549 and Hs-578T (**C**–**D**) cells were treated for 24 h with HTO at the concentrations indicated (A and B) or 200 μM (C and D) and analyzed in immunoblots: Total Akt and phospho-(S473) (p-Akt), total ERK and phospho-ERK (p-ERK) and LC3B-II, and tubulin as a loading control. Protein levels were determined from the immunoreactive bands and the bars correspond to the mean ± SEM values from at least 2 independent experiments with duplicate samples: ^*^p < 0.05, ^***^p < 0.001.

HTO induced heterogeneous changes in lipids that modify the biophysical properties of the cell membrane and that alter cell signaling. We further tested this by analyzing the status of the ERK and Akt signaling proteins. In this context, ERK phosphorylation (activation) was enhanced in BT-549 cells incubated with HTO, which was followed by a rise in LC3B-II, a marker of autophagy that is activated by multiple stressors. Thus, the levels of this protein rose to 128.47% (400 μM) in MDA-MB-231 cells and in BT-549 cells, which was associated with the LC3BI/LC3BII ratio induced by HTO treatments (Figure 4C–4D). These results were consistent with the response to HTO reported previously in NSCLCs [30]. No significant changes were observed in Hs-578T cells exposed to the concentrations HTO used here, in accordance with its failure to alter proliferation at the concentrations studied (Figure 4C–4D). By contrast, we observed significantly weaker Akt and ERK activation in MDA-MB-231 cells exposed to HTO for 24 h (Figure 4A–4B). Moreover, the PARP levels of these cells remained unchanged, indicating only a minor influence of apoptosis (Supplementary Figure 2). These heterogeneous results in part explain the different antitumor effects of HTO, which might reflect its distinct manner of regulating membrane lipids and cell signaling.

### HTO does not change the distribution of EGFR in membrane liquid ordered domains (lipid rafts) but it does enhance EGFR internalization

**Figure 5:**
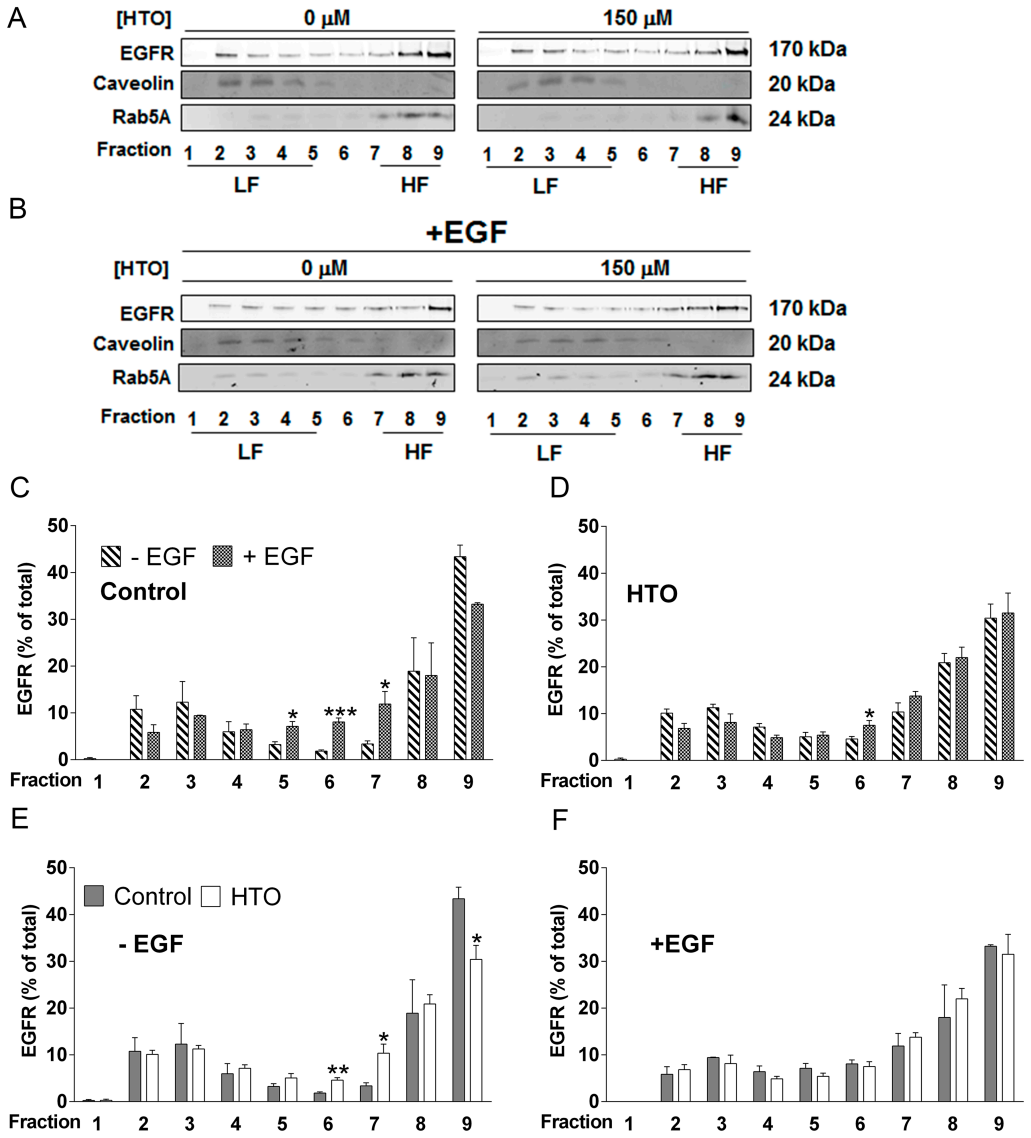
Effect of HTO on the membrane microdomain distribution of EGFR (**A**–**F**) MDA-MB-231 cells were serum starved and cultured for 24 h in the presence or absence of HTO (150 μM), before they were exposed to EGF (100 ng/ml) or the vehicle alone for 30 min. The cells were collected, solubilized in 1% Brij 98 polyoxyethylene fatty ether detergent and subjected to sucrose gradient separation before analyzing the EGFR distribution in detergent-resistant heavy (HF) and light (LF) membrane fractions in western blots (identified by Rab5 and Caveolin, respectively). Representative western blots are shown and the bars correspond to the mean ± SEM values of 2 independent experiments: ^*^p < 0.05, ^**^p < 0.01, ^***^p < 0.001 vs control.

EGFR is a RTK that is overexpressed in some TNBC cells and which modulates Akt, MEK and other signaling pathways [31–33]. EGFR activity has been implicated in proliferation, migration and differentiation of normal cells but also, in the malignant transformation of some tumor cells. When the effects of membrane localization of EGFR into lipid rafts has been studied, both a downregulation of EGFR activation [34, 35] and enhanced EGFR signaling [36] have been described. Here we assessed how HTO (150 μM) affects the nanodomain localization of EGFR and the activation of EGFR in MDA-MB-231 cells. Lipid raft analysis of treated and control cells showed how HTO affects the distribution of EGFR into the different fractions collected by centrifugation. Both EGF (Figure 5C) and HTO (Figure 5E) induced similar changes in the distribution of EGFR in distinct membrane microdomains, although EGF stimulation of HTO-treated cells did not significantly alter the localization of EGFR in the membrane (Figure 5F). This result suggests that HTO provokes a redistribution of EGFR in the membrane of MDA-MB-231 cells similar to that induced by EGF (Figure 5).

**Figure 6:**
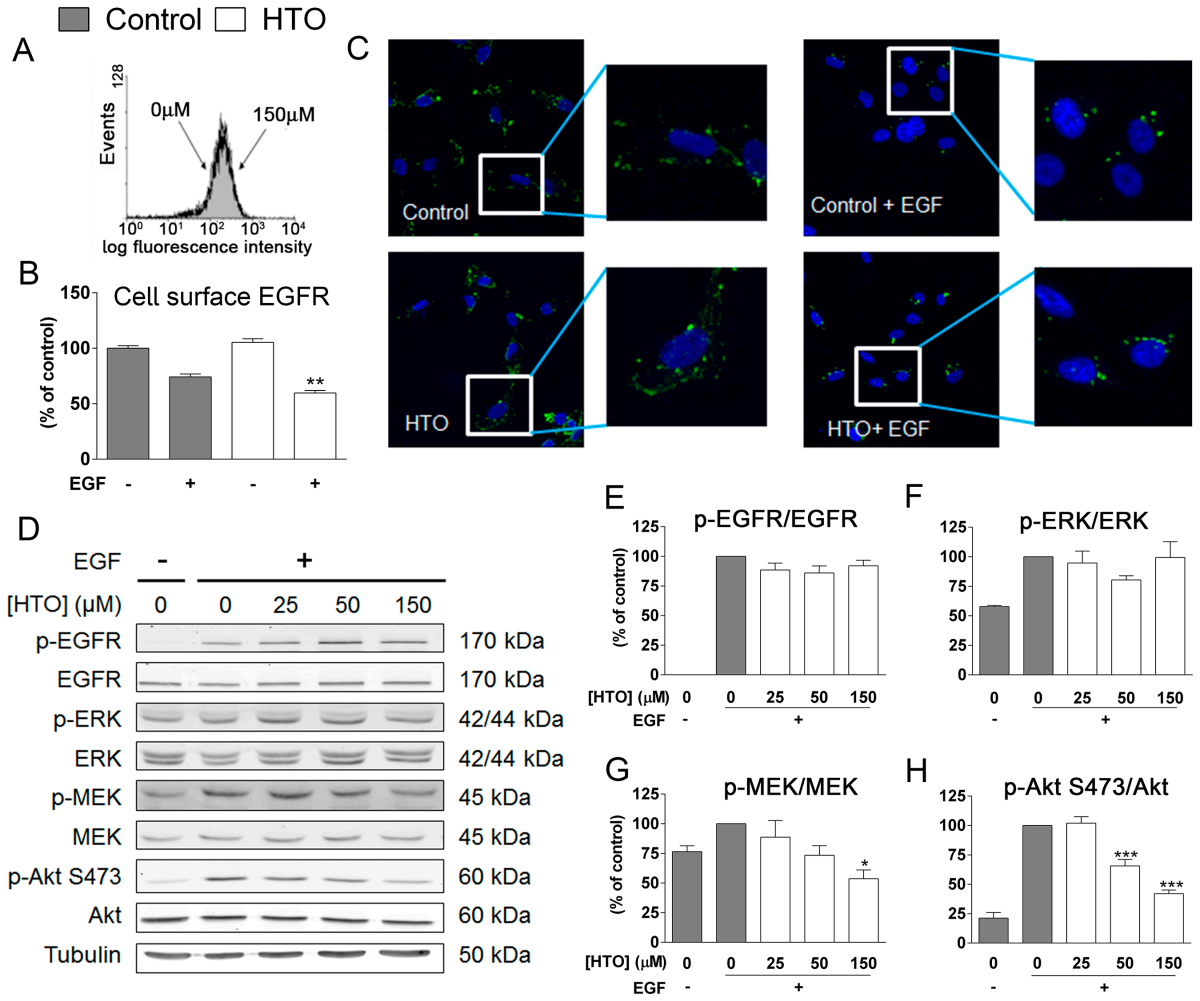
The effect of HTO on EGFR signaling MDA-MB-231 cells were serum starved, treated with HTO (150 μM) for 24 h and stimulated for 30 min with EGF (100 ng/ml) as appropriate. (**A**–**B**) Flow cytometry analysis of EGFR at the MDA-MB-231 cell surface. (A) Expression of EGFR at the cell surface was measured by the intensity of the FITC fluorescence. (B) Internalization of EGFR was measured as the decrease of EGFR at the cell surface. (**C**) EGFR (green) localization by confocal microscopy, identifying the cell nuclei with DAPI (blue). The boxes indicate the regions magnified. (**D**–**H**) Quantitative immunoblotting of relevant signaling proteins in representative western blots. The bars correspond to the mean ± SEM values of 3 independent experiments with duplicate samples, and α-Tubulin was used as a loading control: ^*^p < 0.05, ^***^p < 0.001.

Similarly, as HTO did not produce a significant change in EGFR levels at the cell surface in TNBC cells, as determined by flow cytometry, we studied EGFR activation (dimerization followed by autophosphorylation Figure 6A). After EGF stimulation, there was a loss of cell surface EGFR as a consequence of internalization, and HTO pre-treatment (150 μM) followed by EGF stimulation provoked stronger internalization relative to untreated cells (Figure 6B–6C). However, the phosphorylation of EGFR and hence, its activation was similar in cells grown in the presence or absence of HTO (Figure 6D–6E). Receptor endocytosis has been linked to both signaling attenuation and signaling enhancement. To determine the effect of EGFR internalization on cell signaling in these TNBC cells, we studied Akt and MEK/ERK signaling in the presence and absence of HTO, EGF stimulation of EGFR phosphorylation did not significantly alter these signaling pathways. However, the enhanced internalization of EGFR provoked by HTO was paralleled with weaker MEK and Akt phosphorylation (Figure 6D and 6F–6H).

### HTO increases phosphate turnover

**Figure 7:**
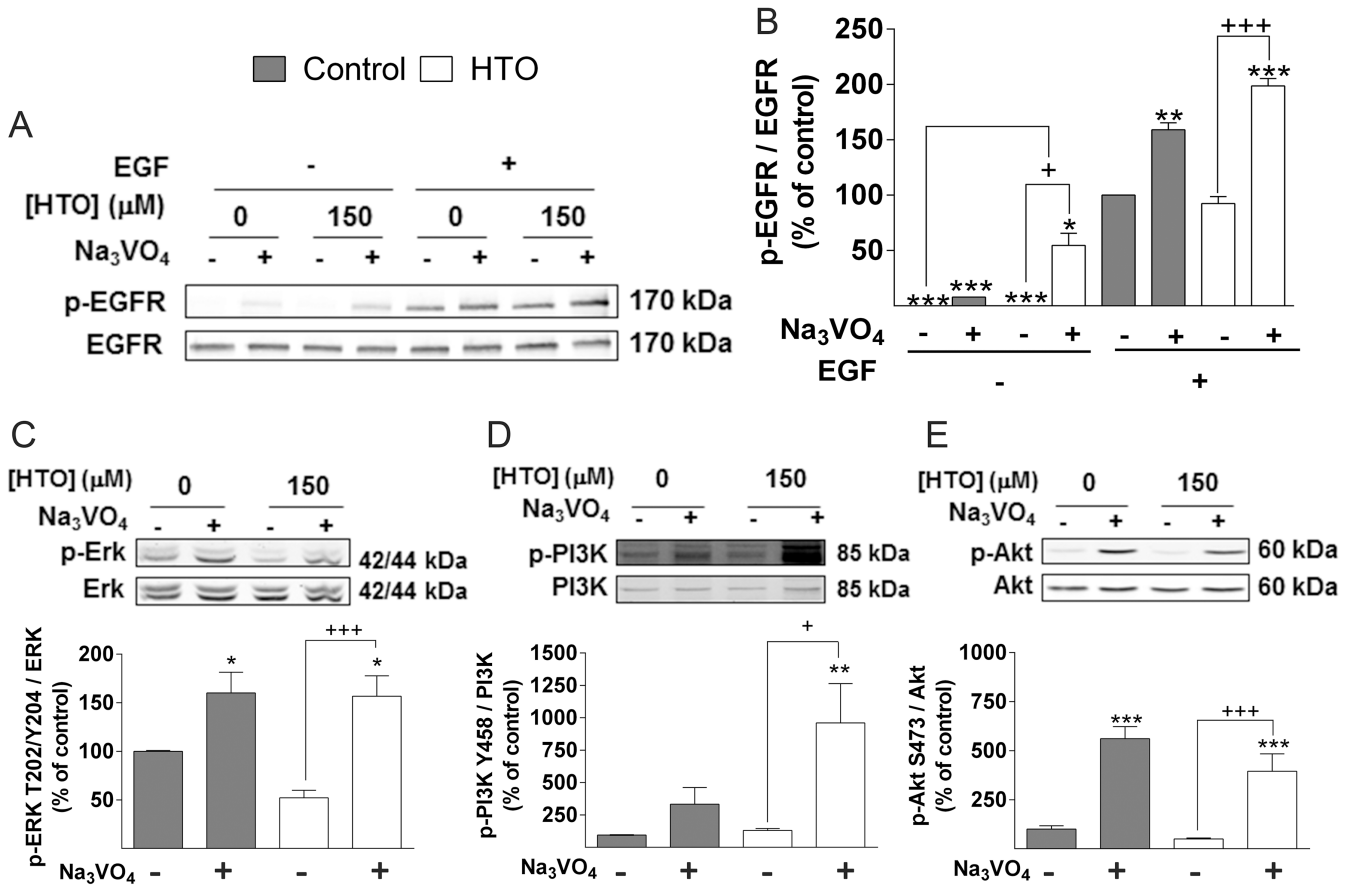
Effect of HTO on phosphatases MDA-MB-231 cells were serum starved, cultured in the presence or absence of HTO (150 μM) for 24 h and orthovanadate (100 nM) for 2 h before the cell lysates were prepared for immunoblotting (**A**–**E**). Cells were stimulated for 15 min with EGF (100 ng/ml) as appropriate (A-B) and the protein levels were determined by quantitative immunoblotting. Representative western blots are shown and the bars correspond to the mean ± SEM values of 3 independent experiments: ^*/+^*p* < 0.05, ^**^p < 0.01, ^***/+++^*p* < 0.001.

Internalized EGFR can be dephosphorylated by protein tyrosine phosphatases after ligand-receptor dissociation. EGFR activation was enhanced in MDA-MB-231 cells stimulated with EGF in the presence of HTO (150 μM) and the general phosphatase inhibitor, sodium orthovanadate [37] (Figure 7A–7B), in conjunction with an increase in ERK phosphorylation in the presence or absence of EGF (Supplementary Figure 3A–3B). By contrast, EGF stimulation in the presence of orthovanadate did not enhance Akt activation in cells when they were treated with HTO (Supplementary Figure 3C–3D). Akt is activated by phosphatidylinositol 3-kinase (PI3K), a lipid kinase that generates phosphatidylinositol-3,4,5-trisphosphate (PIP3), and that links oncogenes and other receptors to cellular survival, proliferation and differentiation. Cellular levels of PIP3 are tightly regulated by phosphatases and phosphorylases [38, 39, 40], thus we treated cells with sodium orthovanadate to study the influence of HTO on the activation of ERK and PI3K. Phosphatase inhibition caused a huge increase of their activation, which was significantly higher in HTO-treated cells (Figure 7C, 7D and Supplementary Figure 3). This activation preceded the increased phosphorylation of the downstream signaling kinase Akt due to the rise in PIP3 production [41, 42]. However, despite of the stronger activation of PI3K in HTO-treated cells, there was no difference in Akt phosphorylation with respect to control untreated cells (Figure 7D–7E).

### HTO induced ceramide synthesis in MDA-MB-2331 cells

Akt is activated by phosphoinositide-dependent kinase-1 (PDK-1), which phosphorylates its Thr^308^ and mTORC2 (the mTOR/RICTOR complex), which phosphorylates Ser^473^. Conversely, protein phosphatase 2A (PP2A) [43, 44] and phosphatase PHLPP inactivate Akt. Ceramide (Cer) can activate PP2A, which dephosphorylates Thr^308^ and the atypical protein kinase C, PKCζ. Ceramide-activated PKCζ leads to phosphorylation of the PH domain in Akt (Thr34), which inhibits PIP3 binding to Akt and its ensuing phosphorylation [45].

**Figure 8:**
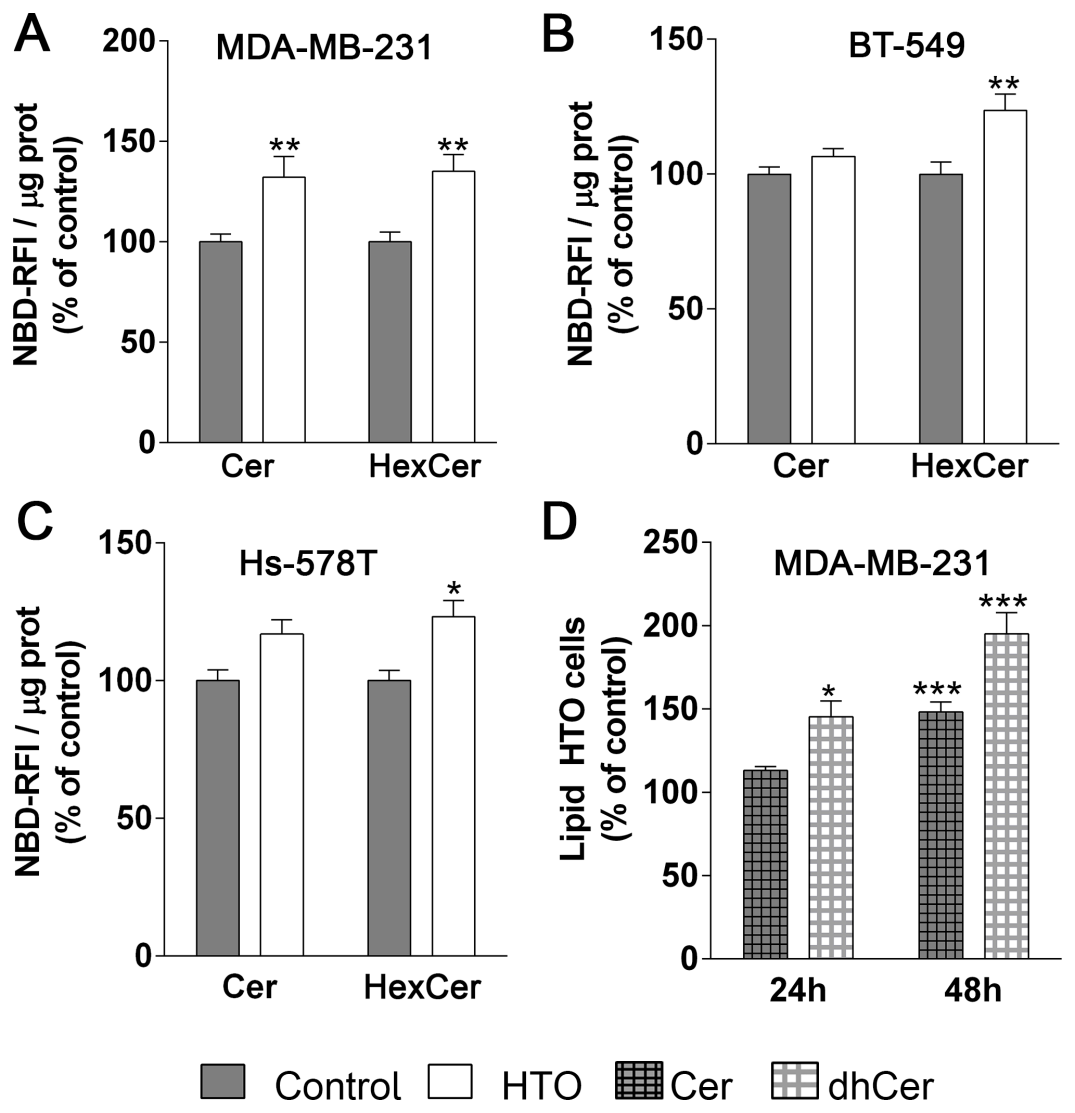
Effect of HTO on ceramide levels TNBC cells (MDA-MB-231, BT-549 and Hs-578T) were pre-treated for 24 h with HTO (200 μM) and then exposed to NBD-sphingomyelin (30 μM) for 3 h. Lipids were separated by TLC, and the plates were developed and scanned to detect and quantify the NBD-containing species. (**A**–**C**) The bars correspond to the mean ± SEM values of 3 independent experiments with duplicate samples: ^*^p < 0.05, ^**^p < 0.01 (ANOVA followed by Bonferroni’s test). (**D**) MDA-MB-231 cells were treated for 24 h with HTO (200 μM) and their lipids were then analyzed by HPLC-MS/MS. The bars correspond to the mean ± SEM values of 2–3 independent experiments: ^*^p < 0.05, ^***^p < 0.001. Abbreviations: Cer, ceramide; HexCer, hexosylceramide; dhCer, dihydroceramide.

Ceramide can be produced by *de novo* synthesis, the salvage pathway or through the sphingomyelinase pathway [46]. When we exposed HTO treated MDA-MB-231 cells to NBD-sphingomyelin, there was an increase in NBD-Cer (32%) and NBD-hexosylceramide (NBD-HexCer, 35%), while HexCer but not Cer levels increased (Figure 8) in BT-549 (24%) and Hs-578T (23%) cells. These results suggest that the sphingomyelinase pathway and Cer glycosylation were more active in MDA-MB231 cells treated with HTO. We also found that Cer and dihydroceramide (dhCer), mainly C16dhCer and C22dhCer, increased in MDA-MB-231 cells treated with HTO (Table 2).

**Table 2A:** Ceramide levels (pmol/mg protein)

FA	24 h		48 h	
	Control	HTO	Control	HTO
**C14Cer**	18.4 ± 5.2	18.3 ± 5.2	27.4 ± 2.9	35.9 ± 2.9
**C16Cer**	561.9 ± 166.3	544.0 ± 119.52	873.5 ± 137.9	1198.5 ± 107.0^*^
**C16:1Cer**	37.2 ± 11.4	37.4 ± 10.3	55.1 ± 5.44	69.1 ± 5.0
**C18Cer**	121.1 ± 66.7	139.5 ± 60.7	274.4 ± 38.8	399.7 ± 35.6
**C20Cer**	40.7 ± 26.6	42.6 ± 24.47	94.4 ± 18.0	135.5 ± 7.8
**C22Cer**	348.9 ± 100.1	429.9 ± 144.07	463.2 ± 43.5	643.8 ± 16.6
**C24Cer**	1842.5 ± 424.9	2135.9 ± 534.47	2095.0 ± 96.6	2732.8 ± 175.3^***^
**C24:1Cer**	440.2 ± 157.8	482.0 ± 123.0	581.9 ± 50.8	906.8 ± 67.4^**^
**C24:2Cer**	104.5 ± 33.0	116.3 ± 32.2	144.45 ± 12.8	205.7 ± 11.4

**Table 2B:** dhCeramide levels (pmol/mg protein)

FA	24 h		48 h	
	Control	HTO	Control	HTO
**C14dhCer**	2.1 ± 0.1	2.5 ± 0.4	5.9 ± 0.5	12.2 ± 2.7
**C16dhCer**	29.1 ± 2.8	38.4 ± 3.3	147.0 ± 13.5	243.1 ± 7.5^***^
**C18dhCer**	10.0 ± 2.4	9.6 ± 0.6	20.4 ± 1.2	36.1 ± 2.9
**C20dhCer**	9.5 ± 0.8	16.2 ± 0.1	34.6 ± 3.9	56.7 ± 1.5
**C22dhCer**	31.0 ± 1.2	39.8 ± 4.4	93.3 ± 11.9	166.6 ± 1.6^***^
**C24dhCer**	109.4 ± 16.0	156.4 ± 14.4^**^	235.8 ± 23.9	597.8 ± 77.0^***^

MDA-MB-231 cells were treated for 24 h with HTO (200 μM) or the vehicle alone and they were then analyzed by HPLC-MS/MS. The values correspond to the mean ± SEM, expressed as pmol lipid/mg protein: ^*^p < 0.01, ^***^p < 0.001. FA, fatty acid; Cer, ceramide; dhCer, dihydroceramide.

### HTO inhibited tumor progression in a xenograft model of human TNBC

**Figure 9:**
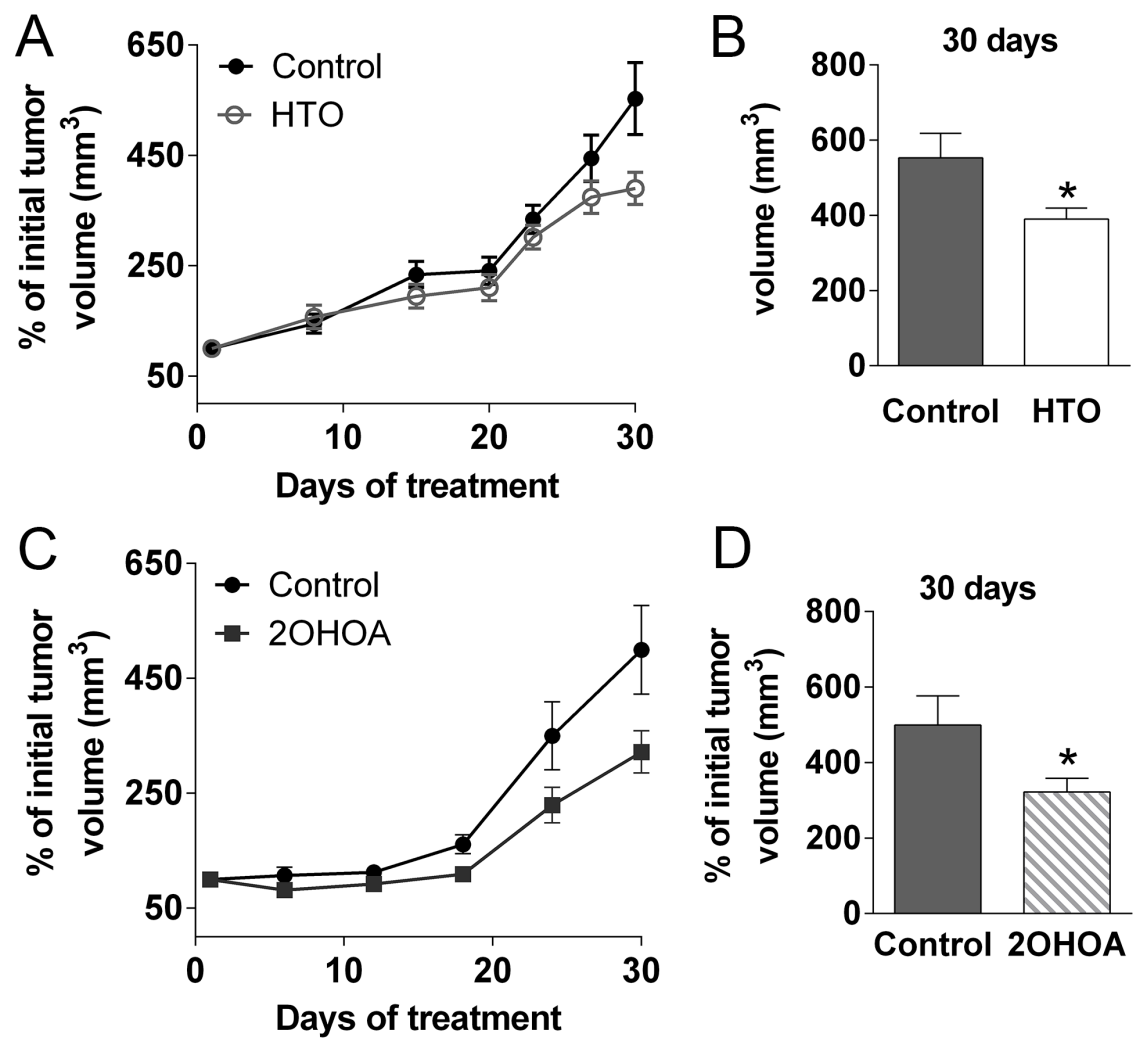
HTO and 2OHOA inhibit tumor progression *in vivo* MDA-MB-231 cells (7.5 × 10^6^) were injected subcutaneously into nude mice, which were then treated orally for 30 days with HTO (400 mg/kg per day -A) or its fatty acid, 2OHOA (C). Tumor size (**A**, **C**) was measured with a digital caliper and the tumor volume was expressed relative to the volume at the beginning of the treatment (mean ± SEM; *n* = 9 for control, *n* = 8 for HTO, *n* = 10 for 2OHOA): ^*^p < 0.05 (Student’s *t* test). Solid circles correspond to the control group, open circles to the HTO group and the solid squares to 2OHOA treatment. (**B**, **D**) The mean tumor size in animals treated with HTO or 2OHOA is compared to the controls (day 30).

The efficacy of HTO against human TNBC cells was tested in nude mice into which MDA-MB-231 cells were grafted. HTO and 2OHOA treatment (400 mg/kg per day, p.o.) induced a marked and significant reduction in tumors relative to untreated mice (Figure 9A–9D). During treatment, no changes in body weight, mortality or other side effects were observed in association with HTO or 2OHOA treatment, which indicated that their oral administration was well tolerated. Moreover, the autopsy of some animals failed to reveal any macroscopic signs of toxicity in the lungs, kidneys, heart or stomach, or fat accumulation in the liver, and normal feces were found in the intestine.

## DISCUSSION

Membranes are exposed to multiple stimuli from circulating cytokines, growth factors, hormones, etc., although only certain cells respond to signals at any specific moment. This is because the receptors that bind these messengers must physically interact with the transducers that propagate these signals in order to produce second messengers. Receptor-transducer interactions require both protein partners to coincide at the membrane, an event that may be regulated by the lipid composition of the plasma membrane. In this context, there are alterations to the lipid composition of membranes in cancer cells that activate cell proliferation. Therapeutic interventions that regulate the lipids in the bilayer can induce the translocation of certain transducers of proliferation to the cytoplasm and impair cell growth [10]. Here, we investigated the effect of the TG, HTO, on membrane lipid composition, cell signaling and tumor growth. We found that the changes in membrane composition provoked by this synthetic lipid induced important alterations in proliferation signals, which induced the cell cycle arrest and cell death that could at least partially explain the tumor regression associated with HTO administration in an animal model of TNBC.

**Figure 10:**
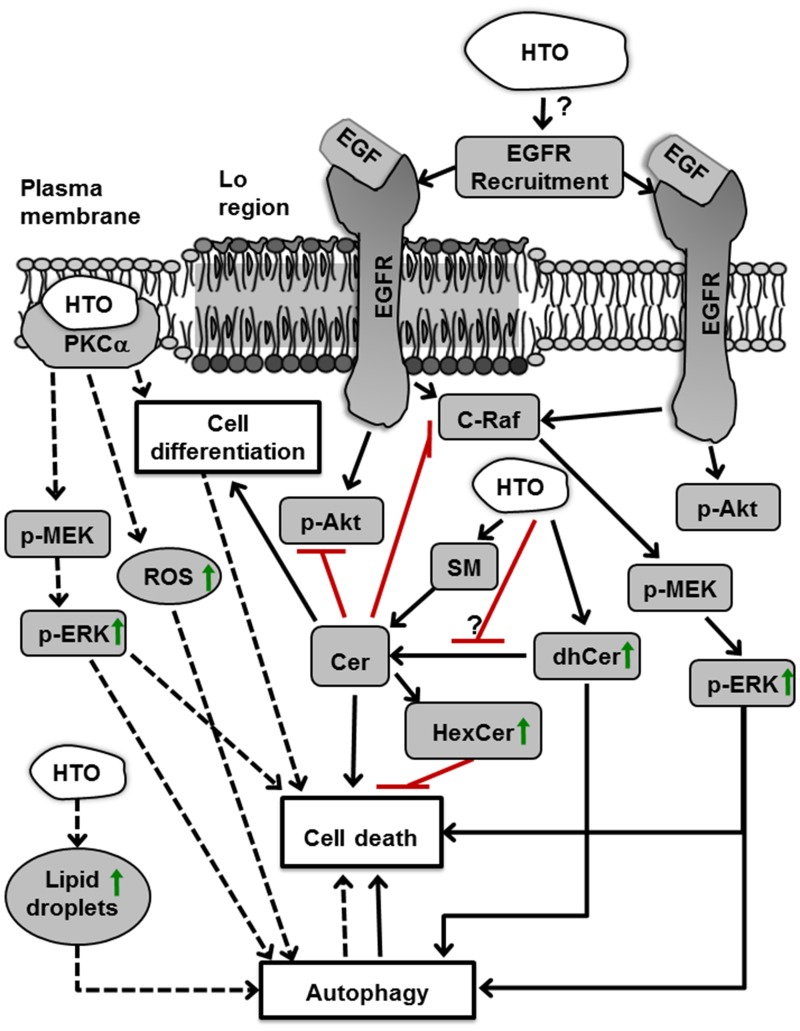
Molecular and cellular effects of HTO HTO activates two pathways in TNBC cells. First, it increases the dhCer levels, which induces cell death by autophagy. In addition, it can increase the amount of Cer, which activates phosphatases and attenuates signaling induced by growth factors, provoking cell death. Moreover, there is an increase in the activity of the enzyme converting sphingomyelin (SM) into Cer (sphingomyelinase), which could also raise the amount of Cer. These and other changes in membrane lipid composition, and the biophysical properties of membranes, induce PKCα translocation to the membrane (i.e., activation), which in turn triggers ERK activation and ROS production, provoking increased autophagy and cell death. HTO also augments lipid droplets in the cells, which also enhances autophagy (dashed line) [30]. Abbreviations: Akt, protein kinase B; Cer, ceramide; C-Raf, rapidly accelerated fibrosarcoma isoform C; dhCer, dihydroceramide; EGF, epidermal growth factor; EGFR, epidermal growth factor receptor; ERK, extracellular-signal regulated kinases; FFA, free fatty acids; HexCer, Hexosylceramide; HTO, hydroxytriolein; Lo, liquid ordered; MEK, mitogen-activated protein/extracellular signal-regulated kinase; PKCα, protein kinase C alpha; ROS, reactive oxygen species; SM, sphingomyelin.

We witnessed here that HTO had a strong antitumor activity against TNBC cells *in vitro* and it prevented their tumor progression *in vivo*. The effects of HTO on TNBC cell signaling and survival depend on the cell type, inducing dhCer and Cer production, and phosphatase or ERK activation, which ultimately led to cell cycle arrest, cell death and/or autophagy (Figure 10). In this context, the stronger antitumor activity of HTO with respect to TO was at least in part related to the different metabolites produced by these compounds [30], which affected the membrane lipid composition distinctly. On the one hand, HTO-treated cell membranes contained unusual fatty acids like 2OHOA (OH-C18:1) and HDA (C17:1), which would correspond to the fatty acyl moiety of HTO and its α-oxidation metabolite, respectively. Hydroxylated fatty acids are naturally present in some tissues and organs, such as the brain, where they are involved in myelination and differentiation, and they are critical to define the sphingolipid profile [47]. In addition, these fatty acids also appear to be enhanced in the glycolipid species found in cancer cell membranes [48]. Moreover, fatty acids that are hydroxylated at C2 make some cancer cells sensitive to the antitumor drug PM02734 [49], and we previously showed that the C2-hydroxylated 2OHOA produces antitumor effects against several types of cancer [8–10]. Within cells, 2-hydroxylated fatty acids are catabolized by the α-oxidation pathway, which yields a fatty acid with one carbon less [50]. Here, we demonstrate that HTO-treated cell membranes contain 2OHOA and HDA, the former with intrinsic antitumor activity. However, the low levels of this fatty acid produced upon HTO treatment are fully consistent with the antitumor effect exerted by this TG. The description here of the distinct effects of HTO and TO on membrane lipids in part explains their different effects on protein-membrane interactions, cell signaling and the inhibition of cancer cell proliferation, as hypothesized by the theory of MLT [51].

We confirm here that HTO and TO also have different effects on TNBC the cell’s lipid composition. TO-treated cells have higher levels of palmitic acid (C16:0), stearic acid (C18:0) and oleic acid (C18:1). This fatty acid accumulation outside of the adipose tissue, especially that of palmitic acid, provokes lipotoxicity, cell dysfunction or death. Different lipotoxic pathways are activated by the distinct fatty acids present in excess in non-adipose tissues [52]. Similarly, changes in the levels of saturated and unsaturated fatty acids influence the cell’s physiology. For example, OA induces cell proliferation and reduces apoptosis, in part due to the upregulation of the TG-FFA (free fatty acid) cycling which sustains glycolysis [53]. Here, TO (which contains OA) caused a decrease in TNBC cell proliferation at the concentrations used, which would in part explain the positive effects of olive oil (rich in TO) against breast cancer [54]. OA, the main fatty acid in TO (100%) and olive oil (70–80%) induces stearoyl-CoA desaturase-1 (SCD1) inhibition, which has been shown to be a relevant anticancer drug target that could in part account for the antitumor effects of TO [12, 55]. As a result of SCD1 inhibition, there is a significant increase in palmitic and stearic acids in HS-578T and MDA-MB-231 cells, whereas increases in OA most likely originate on TO digestion by lipases. Cancer cells have high energy requirements and their fatty acid intake is much faster than that of non-tumor cells [12]. This fatty acid use and the *de novo* synthesis of fatty acids that promote apoptosis after HTO treatment (e.g., palmitate) might be responsible for the programmed cell death of malignant cells. This mechanism of action would be shared by HTO, TO and their fatty acyl derivatives, 2OHOA and OA, which also inhibit SCD1 [12]. Hs-578T cells, which are more resistant to HTO and TO treatment, showed high triacylglycerol accumulation, which may incorporate palmitate and prevent its cytotoxic effects.

The cell membrane oleate-to-stearate ratio regulates the biophysical properties and functions of the plasma membrane, and its alteration can cause apoptosis [56, 57]. Here we report an increase in the unsaturated/saturated fatty acid ratio that is cell line and treatment specific. For example, this ratio increased in MDA-MB-231 cells following exposure to HTO but not TO, whereas the unsaturated/saturated fatty acid ratio in BT-549 cells increased in the presence of TO. This increase induces relevant modifications to the biophysical properties of the plasma membrane, such as its non-lamellar propensity, membrane surface packing density, or lipid raft abundance and dynamics [15]. Moreover, this increase is known to augment membrane fluidity. Regulating the structural properties of the membrane induces important changes in the localization and activity of key membrane proteins that influence cell proliferation and survival, such as Ras or PKC [10, 14].

The molecular events triggered by HTO depend on the cancer cell line, determining its potency and efficacy, as well as the molecular mechanisms that underlie its antitumor activity [14]. Here, HTO activates the ERK pathway and autophagy (as determined by LC3B-II generation) in BT-549 and Hs-578T cells. In NSCLCs, HTO induces autophagy upon PKC activation [30], whereas it inactivates the tumorigenic PI3K/Akt pathway in MDA-MB-231 cells, which may in part explain its antitumor activity. Although NSCLC and MDA-MB-231 cells present molecular and cellular differences, their epithelial origin and cancerous nature could in part explain the similar antiproliferaative effects triggered by HTO. These common antitumor effects are most likely due to similar changes in membrane lipid composition and ensuing regulation of localization and activity of relevant amphitropic cell signaling proteins. This pharmacological mechanism associated with Akt inhibition is consistent with that of 2OHOA (the fatty acid in HTO) in SF767 cells [10], while it differs in HeLa cells [58], suggesting that HTO induces additional antitumor effects to those of its fatty acyl moiety. Moreover, our resulst suggest that Cer but not EGFR signaling is responsible for the decrease in Akt signaling in HTO-treated cells, possibly due to Cer-induced activation of PP2A and PKCζ, and the ensuing impairment of Akt binding to the membrane [45]. OA is the fatty acid in TO, which binds to G protein-coupled receptor 40 (GPR40), the receptor for medium-and long-chain fatty acids, in order to inhibit tyrosine phosphatase 1B (PTP1B) and to stimulate integrin-linked kinase [56, 59, 60, 61]. OA can activate EGFR signaling in endothelial cells [62] and in MCF-7 breast cancer cells [63], yet it does not appear to affect EGFR tyrosine phosphorylation in MDA-MB-231 breast cancer cells [59]. Indeed, EGFR phosphorylation was similar in MDA-MB-231 cells maintained in the presence or absence of HTO. However, EGFR activation was stronger in HTO-treated cells, suggesting that HTO activates EGFR independently of its ligand and in conjunction with phosphatases. The regulation of membrane structure by 2OHOA and the ensuing activation of the Fas receptor (FasR) in a ligand-free manner has been reported elsewhere [9], further demonstrating that important membrane receptors (e.g., EGFR and FasR) can be activated by modifications to the membrane. Indeed, OA induces autophosphorylation of purified EGFR *in vitro* by acting as a mild surfactant [62]. Apart from their ligands, EGFR can be transactivated by several G protein-coupled receptors, which are modulated by the membrane’s lipid composition. EGFR transactivation by receptors occurs by metalloprotease activation that provokes the release of EGF-like ligands [64].

The EGFR is distributed in different membrane microdomains following HTO treatment, which might reflect its activation. Upon activation, the EGFR is internalized by endocytosis [65] and while clathrin-dependent endocytosis (CDE) of EGFR prevails at low EGF concentrations, at high concentrations EGFR can be internalized by both a clathrin-dependent and a clathrin-independent mechanism (CIE, raft/caveolar) [31]. EGFR internalized through CDE is recycled to the plasma membrane, while CIE causes its degradation [66]. CIE has been described in MDA-MB231 cells [67] and at the concentrations used in our study, EGFR was internalized both via processes. Activation by EGF alters the receptor’s distribution in membrane microdomains, both in cells maintained in the presence and absence of HTO. Here, the amount of EGFR in the membrane raft fraction remained relatively constant, possibly due to weak internalization of EGFR from these membrane fractions. In general, exposing MDA-MB-231 cells to HTO induced changes in the EGFR membrane distribution similar to those induced by EGF. Likewise, 2OHOA induces FasR clustering and its ligand-free activation in leukemia cells [9], indicating that this redistribution of EGFR could influence its activation in the absence of agonist ligands in these breast cancer cells.

Cells exposed to HTO stimulate phosphatases by enhancing Cer and dhCer production. Cer is a key molecule in sphingolipid signaling, a second messenger produced in response to several stimuli that include cell stress after antitumor chemotherapy. Cer can be generated by its *de novo* synthesis from palmitate upon sphingomyelinase activation, and through the *salvage pathway*. The regulation of Cer strongly influences cancer cell death and differentiation, which could represent an avenue to develop therapeutic strategies [68]. Our data indicates that enzymes producing Cer from sphingomyelin (*the SMS pathway*) are more active after exposure to HTO, with the Cer produced through this pathway possibly generating sphingosine, ceramide-1 phosphate or HexCer. Endogenous Cer levels might also increase due to poor clearance following inhibition of the glucosylceramide synthase, sphingomyelin synthase (SMS) or ceramidase enzymes. HexCer levels are enhanced after HTO treatment, which protects cells from the pro-apoptotic effects of Cer [69, 70].

We detect dhCer accumulation before that of Cer, which is synthesized through a *de novo* pathway via sphinganine acylation, and that can then be converted into Cer by dihydroceramide desaturases (Des1 or Des2). HTO treatment favors the accumulation of dhCer in cells, indicating enhanced activity of the *de novo* pathway. It was reported that oleate blocks Cer production induced by palmitate, preventing the Des1 up-regulation that results in increased dhCer [71]. Hence, the increase in dhCer after exposure to HTO could reflect overactivation of the *de novo* synthesis pathway, Cer production and/or partial Des1 inhibition by the oleate analogue in HTO, 2OHOA.

It has been indicated that dhCer induces differentiation, and that it regulates apoptosis, cell proliferation and autophagy [72]. The presence of a double bond in Cer but not in dhCer induces biophysical properties that differ from those induced by dhCer in membranes. Therefore, the dhCer/Cer ratio is important to define the biophysical nature of the lipid bilayer and the signaling events it controls, with Des1 inhibition increasing the dhCer/Cer ratio and the rigidity of the plasma membrane [73]. Moreover, the bioactive effects of dhCer depend on the chemical nature of dhCer (e.g., the length and fatty acyl moiety), such that an increase in dhCer C22:0 and dhCer C24:0 in leukemia cells induces cytotoxicity through a caspase-independent mechanism that is associated with autophagy [74]. Because HTO markedly and significantly increased these dhCer species, the antitumor effects of this sphingolipid were most likely associated with the regulation of dhCer C22:0 and C24:0 levels. In addition, Cer-16 and Cer-24 increased in HTO treated cells. Cer has often been proposed to act against cancer, mainly regulating cell survival [75]. The effects of Cer as an inducer of cancer cell death has been proposed as a potential approach for drug discovery [68], although the various forms and effects of these lipids makes this a complex task.

Overall, this study indicates that HTO has a good antitumor potential. Its non-hydroxylated analogue and main triacylglycerol species in olive oil, TO, has a relevant although weaker effect against tumor cells, which may account for the anticancer properties of olive oil. In general, HTO induced similar effects against TNBC cell proliferation to those exerted by TO, although more potently, yet HTO also induces other effects on membrane lipid composition that may explain its stronger pharmacological effect. Thus, HTO causes a dramatic increase in the saturated-to-unsaturated fatty acid ratio, which alters the membrane’s biophysical properties and has toxic effects on cancer cells [80]. In addition, HTO causes marked increases in dhCer and Cer, and in acyl glycerol, which justifies the translocation of PKC to membranes and its activation [81, 82], promoting the signaling events that impair TNBC cell and tumor growth. These lipid alterations affect the interactions of other pivotal signaling proteins with membranes, modifying cell signaling and triggering specific cancer cell death [81].

HTO seems to be a 2OHOA reservoir, although its different molecular structure would allow additional effects to those found in the fatty acid, which suggests that HTO could be used to treat different types of cancer. Currently, 2OHOA has successfully terminated a phase I/IIA clinical trial in patients with advanced gliomas and other solid tumors, showing therapeutic benefit in almost half of the patients, who were refractory to other treatments. A pivotal phase IIB study in patients with glioblastoma multiforme has been initiated to further demonstrate the efficacy and safety of 2OHOA, and a pediatric trial in patients with neurological tumors will start soon. Upon termination of the phase IIB study, the EMA will grant marketing authorization if 2OHOA shows similar efficacy and safety to those observed in the phase I/IIA trial in patients treated for 6 weeks or more, and demonstrates statistical improvement with respect to the current standard of care for this devastating condition. The clinical investigation results obtained hitherto support the great potential of hydroxylated lipids against cancer. In this scenario, the present study sheds light on the mechanism of action of HTO and it further defines the membrane lipid bilayer as a relevant target to combat TNBC, and other types of cancer [82].

## MATERIALS AND METHODS

### Reagents and antibodies

HTO was obtained from Medalchemy (Alicante, Spain). The RPMI medium, triolein, RNaseA and propidium iodide were purchased from Sigma-Aldrich (St. Louis, USA), with penicillin-streptomycin purchased from Biowest (Nuaillé, France) and the fetal bovine serum (FBS) from Biosera (Boussens, France). The recombinant human EGF was obtained from R&D systems (Minneapolis, USA), while sodium orthovanadate was from Sigma-Aldrich (St. Louis, USA) and the protease inhibitors were from Roche (Roche, Basel, Switzerland). NBD-sphingomyelin and NBD-glucosylceramide were purchased from Larodan (Solna, Sweden), and NBD-ceramide from Invitrogen, Molecular Probes. The antibodies used to probe Western blots were raised against LC3BI-II, ERK, phospho-ERK (p-ERK), Akt and phospho-Akt (p-Akt S473), and they were purchased from Cell Signaling (Danvers, MA, USA). The antibody against α tubulin was purchased from Sigma-Aldrich (St. Louis, USA) and the antibodies against EGFR and p-EGFR (Y1068) were from Abcam (Cambridge, UK). The anti-EGFR antibody 930 used to stain the cell surface and to assess internalization was kindly provided by Genentech (San Francisco, USA).

### Cell lines and culture

The MDA-MB-231, BT-549 and Hs578T TNBC cell lines were obtained from the American Type Culture Collection (Manassas, USA). Cells were incubated at 37°C in a humidified atmosphere of 5% CO_2_ in RPMI supplemented with 10% FBS (v/v), 100 units/mL penicillin and 0.1 μg/mL streptomycin.

### Cell proliferation assays

Metabolic active cells were analyzed using the cell proliferation kit II from Roche, following the manufacturer’s instructions. Briefly, cells were seeded in 96-well plates at a density of 4 × 10^3^ cells per well 12–24 h before treatment, and they were then cultured in the presence or absence of HTO or TO at the concentrations and for the periods indicated in the figures. After different periods, the viable cells in the plate were measured using XTT [sodium 3′-[1-(phenylaminocarbonyl)-3,4-tetrazolium]-bis (4 methoxy-6-nitro)benzene sulfonic acid hydrate) 5-dimethylthiazol-2-yl]-2, 5- diphenyltetrazoliumbromide] with PMS (N-methyl dibenzopyrazine methyl sulfate), adding this to the cell culture medium according to the manufacturer’s instructions (Roche, Basel, Switzerland). The cells were then incubated at 37°C in 5% CO_2_ until a constant color developed and the absorbance was recorded at 495 nm using a microplate reader with a reference wavelength of 650 nm (FLUOstar Omega, BMG LABTECH, Germany).

### Lactate dehydrogenase release

The release of lactate dehydrogenase (LDH) from cells was measured with the LDH cytotoxicity detection kit (Roche, Basel, Switzerland), according to the manufacturer’s instructions. Briefly, cells were cultured in 96-well plates at a density of 3 × 10^3^ cells per well, in the presence or absence of HTO, and at the concentrations indicated in the figures. After 24 h, 2.5% lysis buffer was added to some of the cultures to assess the maximum LDH release. The medium from these cultures and from the other cultures to which lysis buffer was not added was then collected, the LDH reagent was added and the mixture was incubated at room temperature in the dark until a constant color developed. Absorbance was determined at 492 nm on a microplate reader (FLUOstar Omega, BMG LABTECH, Germany) and the test medium was used as a background control. Cytotoxicity was analyzed by comparison to the control cells using the following equation: (HTO treated-untreated)/(maximum LDH-untreated).

### Cell cycle analysis

Cells were cultured in 10 cm diameter culture plates at a density 3.5 × 10^5^ cells per plate. After incubation in the presence or absence of HTO at the concentrations and for the times indicated in figures, the cells were harvested by trypsinization, fixed in ice-cold 70% ethanol, washed with 38 mM sodium citrate (pH 7.0) and stained for 20 min at 37°C with a solution containing 69 μM propidium iodide, 38 mM sodium citrate and 5 μg per ml RNaseA. The cells were subsequently analyzed by flow cytometry on an EPICS XL-MCL flow cytometer (Beckman Coulter, Miami, USA), with excitation at a wavelength of 488 nm.

### Electrophoresis, immunoblotting and protein quantification

Cells were cultured in 10 cm diameter culture plates at a density 3.5 × 10^5^ cells per plate. After incubation in the presence or absence of HTO at the concentrations and times indicated in figures, 300 μl of protein extraction buffer was added to each plate (10 mM Tris-HCl [pH 7.4] containing 50 mM NaCl, 1 mM MgCl_2_, 2 mM EDTA, 1% SDS, 5 mM iodoacetamide and 1 mM PMSF). Cell suspensions were subjected to ultrasound at 50 W for 10 s using a Braun Labsonic U (probe-type) ultrasound homogenizer and 30 μl aliquots were taken for protein quantification using a modified Lowry assay, according to the manufacturer’s instructions (Bio-Rad, California, USA). Samples were prepared for electrophoresis by boiling for 3 min in 10 x electrophoresis loading buffer (120 mM Tris-HCl [pH 6.8], 4% SDS, 50% glycerol, 0.1% bromophenol blue and 10% β-mercaptoethanol). For immunoblotting, total protein (30–50 μg) from the cell lysates was resolved by SDS polyacrylamide gel electrophoresis (SDS-PAGE) and transferred to nitrocellulose membranes (Schleicher & Schüell). After immunoblotting, nitrocellulose membranes were blocked for 1 h at room temperature in PBS (phosphate buffered saline) containing 5% non-fat dry milk and 0.1% Tween 20 (blocking solution). The membranes were then probed overnight at 4°C with the primary antibody in blocking solution at the concentration recommended by the manufacturer. The membranes were then washed three times for 5 min with PBS and incubated for 1 h at room temperature in fresh blocking solution containing the donkey anti-mouse or donkey anti-rabbit IRDye^®^800CW antibody (1:5,000 dilution). Antibody binding was assessed by scanning the membrane with the Odyssey infrared imaging system (Li-COR, Nebraska, USA), determining the α-Tubulin content in the samples using the same procedure and as a loading control. The intensity of the signal was quantified using Total Lab software (Nonlinear Dynamics Ltd).

### Confocal microscopy

Cells were grown in 8-well Millicell ez slide culture plates (Millipore, USA) and treated for 24 h with HTO (150 μM) in serum-free medium containing 1% BSA (bovine serum albumin) prior to stimulation for 30 min with EGF (100 ng/ml). The cells were then washed and fixed with 4% paraformaldehyde for 20 min at RT, washed again with PBS and incubated overnight at 4°C with an anti-EGFR IgG. Finally, the cells were washed with PBS and incubated at room temperature with alexa^®^ 488 conjugated anti-mouse IgG (Invitrogen, Thermo fisher scientific Inc, Waltham, MA, USA) for 1 h. The nuclei were stained with Hoechst (Thermo-scientific, USA) and the cells were visualized on a Leica TCS SPE inverted confocal microscope (Barcelona, Spain).

### EGFR internalization

MDA-MB-231 cells were treated with EGF in the presence of absence of HTO (150 μM) in serum-free medium containing 1% BSA for 24 h and then with EGF. The cells were then collected by trypsinization, incubated at 4°C with an anti-EGFR IgG for 30 min, washed with PBS containing 1% BSA to remove the unbound anti-EGFR, and incubated for 30 min at 37°C with EGF (100 ng/ml). Activation was stopped by placing the cells at 4°C and then washing with PBS containing 1% BSA to remove the unbound EGF. The cells were then incubated with an Alexa^®^ 488 conjugated anti-mouse IgG (Invitrogen, Thermo fisher scientific Inc, Waltham, MA, USA), they were washed and then immediately analyzed on an EPICS XL-MCL (Beckman Coulter, USA) flow cytometer, using 488 nm and 519 nm as excitation and emission wavelengths, respectively. The degree of internalization corresponded to the decrease in the surface EGFR bound.

### Separation of detergent-resistant membranes

The post-nuclear supernatant from 5 × 10^6^ MDA-MB231 cells was solubilized for 1 h on ice in 325 μl of buffer A (25 mM HEPES, 150 mM NaCl, 1 mM EGTA, 10 mM sodium pyrophosphate, 10 mM sodium fluoride, 5 mM orthovanadate, protease inhibitor cocktail) containing 1% Brij 98 (Aldrich, St Louis, MO, USA), followed by the addition of 2 ml of 2 M sucrose in buffer A, before placing it at the bottom of a step sucrose gradient in buffer A (1.33-0.9-0.8-0.75-0.7-0.6-0.5-0.4-0.2 M). The gradient was centrifuged at 4°C and 250,000 × g for 16 h using a SW60Ti rotor (Beckman Coulter Optima L-100 XP ultracentrifuge, Brea, California). Fractions of 325 μl were harvested from the top of the gradient, except for the last fraction that contained 975 μl.

### Lipid analysis

TNBC cells were seeded in 10 cm diameter culture plates at a density of 4 × 10^5^ cells/plate, and maintained in the presence or absence of HTO for 24 h. Lipids were extracted directly from lysates of 1 × 10^6^ cells using the chloroform:methanol extraction method [76] and the protein levels were measured using a modified Lowry assay (Bio-Rad, USA). Individual lipids classes were separated by TLC on Whatman silica gel-60 plates (20 × 20 cm) using petroleum ether/diethyl ether/acetic acid (75:25:1.3 by volume) for neutral lipids and chloroform/methanol/acetic acid/water (60:50:1:4 by volume) for phospholipids. After TLC separation, the plates were air-dried, sprayed with 8% (w/v) H_3_PO_4_ containing 10% (w/v) CuSO_4_, and charred at 180°C for 10 min. The lipids were then quantified by image analysis using Quantity One software (Bio-Rad), and the lipid fractions were identified using 1,2 diolein, oleic acid, triolein, phosphatidyl choline, phosphatidyl serine, phosphatidyl inositol, phosphatidyl ethanolamine and sphingomyelin as standards.

### Fatty acid analysis by gas chromatography (GC)

Extracted lipids were transmethylated by incubating the lipid mixture in 3 ml methanol:acetylchloride (10:1, v:v) for 90 min at 100°C under an argon atmosphere in pyrex screwed-capped tubes [77]. The resultant fatty acid methyl esters (FAMEs) were extracted with hexane, adding 3 ml H_2_O and 1 ml hexane to the transmethylation reaction and thoroughly vortexing the mixture. After centrifugation at room temperature (1,000 × g for 10 min), the upper phase containing FAMEs was collected and the remaining volume was washed twice with 1 ml hexane, repeating this process twice. The hexane phases were combined, evaporated under argon flow and resuspended in 1 ml of hexane. We retained 500 μl of this suspension for direct GC analysis and the remaining volume was evaporated to derive the free hydroxyl groups. For hydroxy fatty acid quantification, a second derivatization with trimethylsilyl was performed [78]. The lipid film was dissolved in N,O-bis (trimethylsilyl) acetamide (0.1 – 5.0 mg lipid for 200–400 μl trimethylsilylation reagent) and heated in a capped vial at 70°C for 30 min. The solvent was evaporated and the lipid film was resuspended in 500 μl hexane.

Derivatized fatty acids were subjected to methylation or to methylation/trimethylsilylation, and analyzed on an Agilent 7890A GC system equipped with a FID and a 7693 auto-injector (Santa Clara, CA, USA). An Agilent J&W HP-88 capillary column (30 m × 0.25 mm × 0.20 μm) was used with 1.3 ml/min of helium as a carrier and the split ratio was 5:1. For GC separation, the column was equilibrated at 130°C for 5 min upon sample injection, before increasing the temperature to 160°C at 2.5°C/min and then to 220°C at 2°C/min. Finally, the column was kept at 220°C for 5 minutes. The injector and detector temperatures were maintained at 250°C, and the areas under the peaks were quantified using margaric acid as an internal standard and corrected using the protein content. Peaks were identified using standards for the different hydroxylated and non-hydroxylated fatty acids. A linear correlation between the peak area and the concentration of fatty acid injected for GC was observed (Supplementary Figure 4).

### SMS activity in cell cultures

SMS activity was determined as described elsewhere [79]. Briefly, TNBCs were incubated for 4 h with NDB-Sphingomyelin (3 μM) and NBD-lipids were separated by TLC. A first solvent system of chloroform/ethanol/water/triethylamine (35:40:9:35, by volume) was used and when the solvent front reached two-thirds of the plate, they were dried and subsequently separated using isohexane/ethylacetate (5:1, by volume) until the solvent front reached the end of the plate. The plates were then dried and the lipids were visualized on a Bio-Rad Molecular Imager FX and quantified using Quantity One software (Bio-Rad).

### Analysis of ceramide and dihydroceramide

Cells were treated in the presence or absence of HTO (150 μM or 300 μM) for the times indicated, collected and homogenized as indicated above, and analyzed at the Lipidomic’s service of the Biomedical Chemistry department at the Institute of Advanced Chemistry of Catalonia.

### Animals, tumor xenografts and treatments

Male NUDE (Swiss) Crl:NU (Ico)-Foxn1^nu^ mice (five week-old, 30–35 g: Charles River Laboratories, Paris, France) were maintained in a thermostat cabinet (28°C: EHRET, Labor-U-Pharmatechnik) with a sterile air flow at a relative humidity of 40–60% and on a 12 h dark/light cycle. Autoclaved food and water were supplied *ab libitum*. To instigate xenograft tumors, 7.5 × 10^6^ MDA-MB-231 cells were subcutaneously inoculated into one side of the animal’s dorsal flank and tumors became visible after one week, with a volume of ca. 100 mm^3^. Animals were divided randomly into groups with a similar mean tumor volume and they received daily p.o. treatments for 29 days with the vehicle alone (water), HTO (400 mg/kg) or 2OHOA. Tumor volumes (*v*) were calculated as *ν* = *W*^2^ × *L*/2, where *w* is the tumor width and *L* is its length. All experiments were carried out in accordance with the animal welfare guidelines of the European Union and the Institutional Committee for Animal Research of the University of the Balearic Islands.

### Data analysis

Statistical analyses were performed using GraphPad Prism 4.01 software (GraphPad Software Inc., San Diego, USA). Unless otherwise indicated, the data are expressed as the mean ± SEM of at least three independent experiments with duplicate samples. Experimental groups were compared using one-way ANOVA followed by the Bonferroni multiple-comparison test. The differences between the experimental groups were considered statistically significant at *P* < 0.05: ^*^P < 0.05, ^**^P < 0.01, and ^***^P < 0.001.

## SUPPLEMENTARY MATERIALS FIGURES AND TABLES



## Abbreviations

2OHOA: 2-hydroxyoleic acid
Akt: protein kinase B
BSA: bovine serum albumin
Cer: ceramide
CDE: clathrin-dependent endocytosis
CIE: clathrin-independent endocytosis
Chol: cholesterol
Des: dihydroceramide desaturase
dhCer: dihydroceramide
ERK: extracellular signal-regulated kinase
EGF: epidermal growth factor
EGFR: epidermal growth factor receptor
FA: fatty acid
FAMEs: fatty acid methyl esters
FasR: Fas receptor
FBS: fetal bovine serum
FFA: free fatty acids
GC: gas chromatography
HDA: heptadecenoic acid
HTO: hydroxytriolein
HexCer: hexosylceramide
HER-2: human epidermal growth factor receptor 2
LDH: lactate dehydrogenase
LC: liquid chromatography
MAPK: mitogen-activated protein kinase
MEK: mitogen-activated protein/extracellular signal-regulated kinase kinase
MLT: membrane-lipid therapy
NSCLC: non-small cell lung cancer
MUFA: mono-unsaturated fatty acid
OA: oleic acid
PBS: phosphate buffered saline
PDK-1: phosphoinositide-dependent kinase-1
PI3K: phosphatidylinositol 3-kinase
PIP3: phosphatidylinositol-3,4,5-trisphosphate
PKC: protein kinase C
PP2A: protein phosphatase 2A
PMS: N-methyl dibenzopyrazine methyl sulfate
RTK: receptor tyrosine kinase
ROS: reactive oxygen species
PC: phosphatidyl choline
PE: phosphatidyl ethanolamine
PI: phosphatidyl inositol
PS: phosphatidyl serine
SFA: saturated fatty acid
SM: sphingomyelin
SCD1: stearoyl-CoA desaturase-1
TLC: thin layer chromatography
TNBC: triple negative breast cancer
TGAs: triacylglycerides
TG: triacylglycerol
TO: triolein
UFA: unsaturated fatty acid
XTT: sodium 3’-[1-(phenylaminocarbonyl)-3,4-tetrazolium]-bis (4 methoxy-6-nitro) benzene sulfonic acid hydrate) 5-dimethylthiazol-2-yl]-2, 5- diphenyltetrazoliumbromide.
