# Supplementary material for: The triacylglycerol, hydroxytriolein, inhibits triple negative mammary breast cancer cell proliferation through a mechanism dependent on dihydroceramide and Akt
Source: Oncotarget. 2019 Apr 2;10(26):2486–507. doi: 10.18632/oncotarget.26824 (PMC6493458; doi:10.18632/oncotarget.26824)
Supplement: Supplementary file 1 [file oncotarget-10-2486-s001.pdf]

The triacylglycerol, hydroxytriolein, inhibits triple negative mammary breast cancer cell proliferation through a mechanism dependent on dihydroceramide and Akt

SUPPLEMENTARY MATERIALS

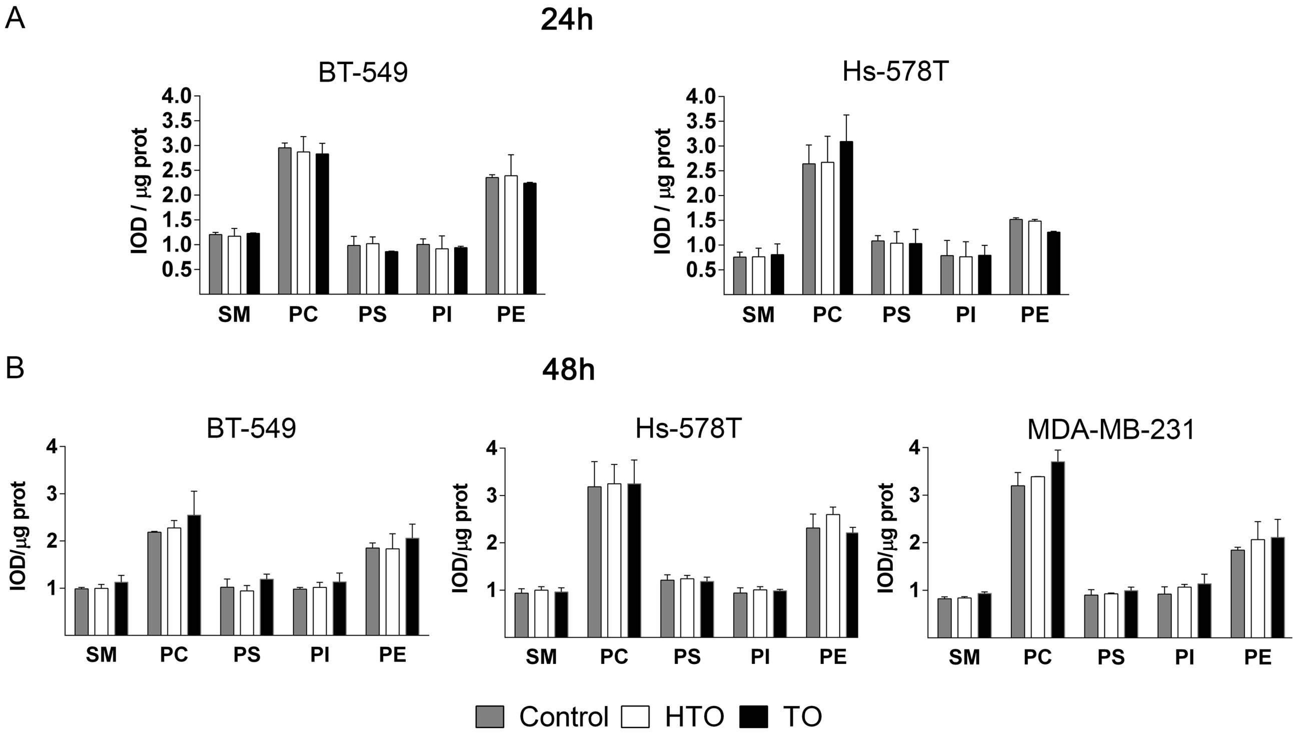

Supplementary Figure 1: HTO and TO affects the phospholipid composition of TNBC cell lines. TNBC cell lines (MDA-MB-231, BT-549 and Hs-578T) were cultured for 24 h or 48 h in the presence or absence (grey bars) of HTO (300 µM, open bars) or triolein (black bars), before the lipids were extracted and fractionated by TLC to measure the phospholipids. The bars correspond to the mean ± SEM values of 2 independent experiments: $^{**}p < 0.01$, $^{***}p < 0.001$ vs control (ANOVA followed by Bonferroni's test). Abbreviations: PC, phosphatidylcholine; PE, phosphatidylethanolamine; PI, phosphatidylinositol; PS, phosphatidylserine; SM, sphingomyelin.

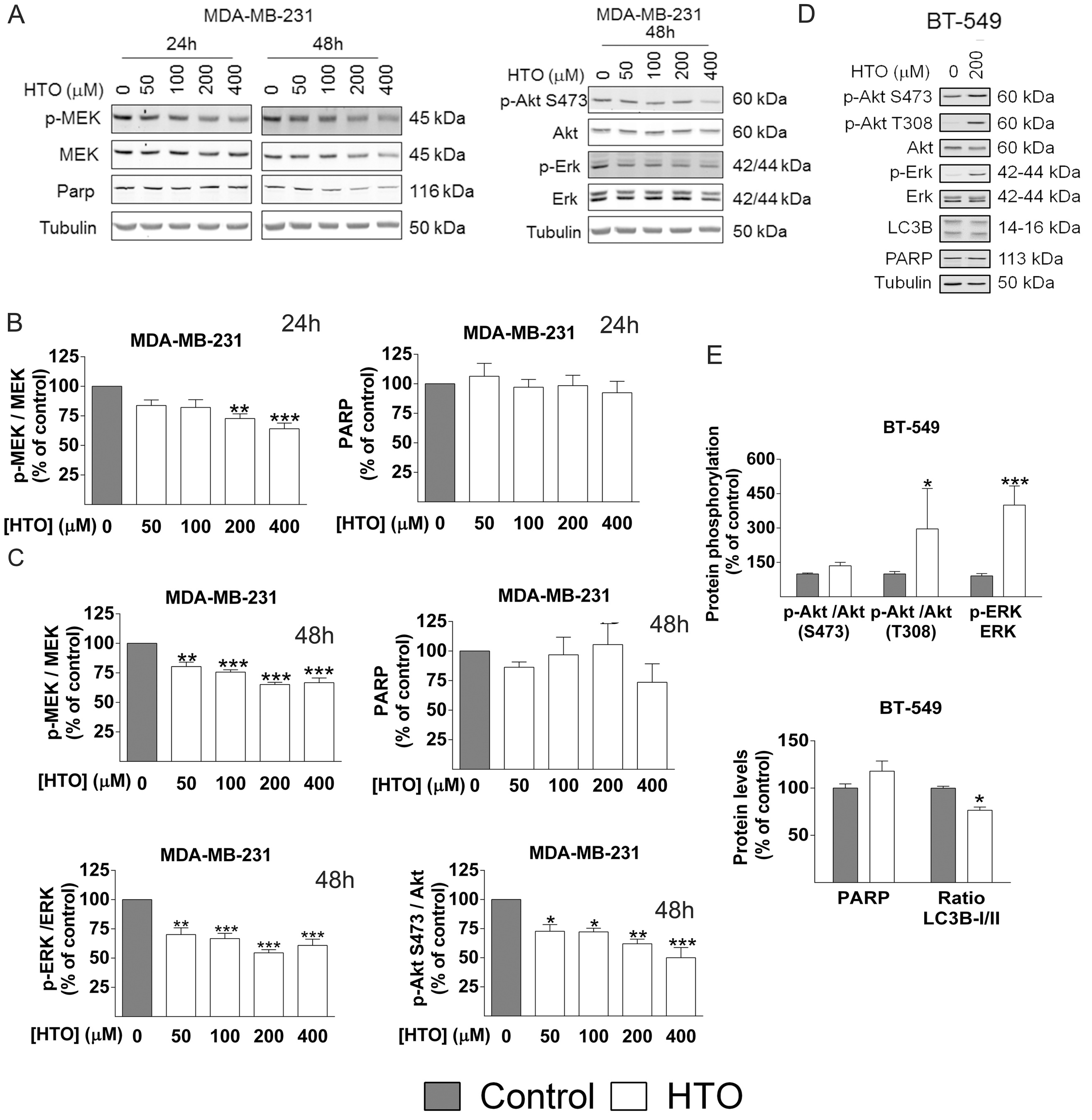

Supplementary Figure 2: Effect of HTO on signaling proteins after a 48 h treatment. (**A–C**) MDA-MB-231 cells were treated for 48 h with the concentrations of HTO indicated and different signaling proteins were quantified in immunoblots. Representative immunoblots are shown and the bars correspond to the mean ± SEM values of 3 independent experiments with duplicate samples. (**D–E**) BT-549 cells were cultured in the presence or absence of HTO (200 μM) for 48 h and signaling proteins were assessed in immunoblots. Representative immunoblots are shown and the bars correspond to the mean ± SEM values of 2 independent experiments with duplicate samples: $^{*}p < 0.05$, $^{***}p < 0.001$ (ANOVA followed by Bonferroni's test).

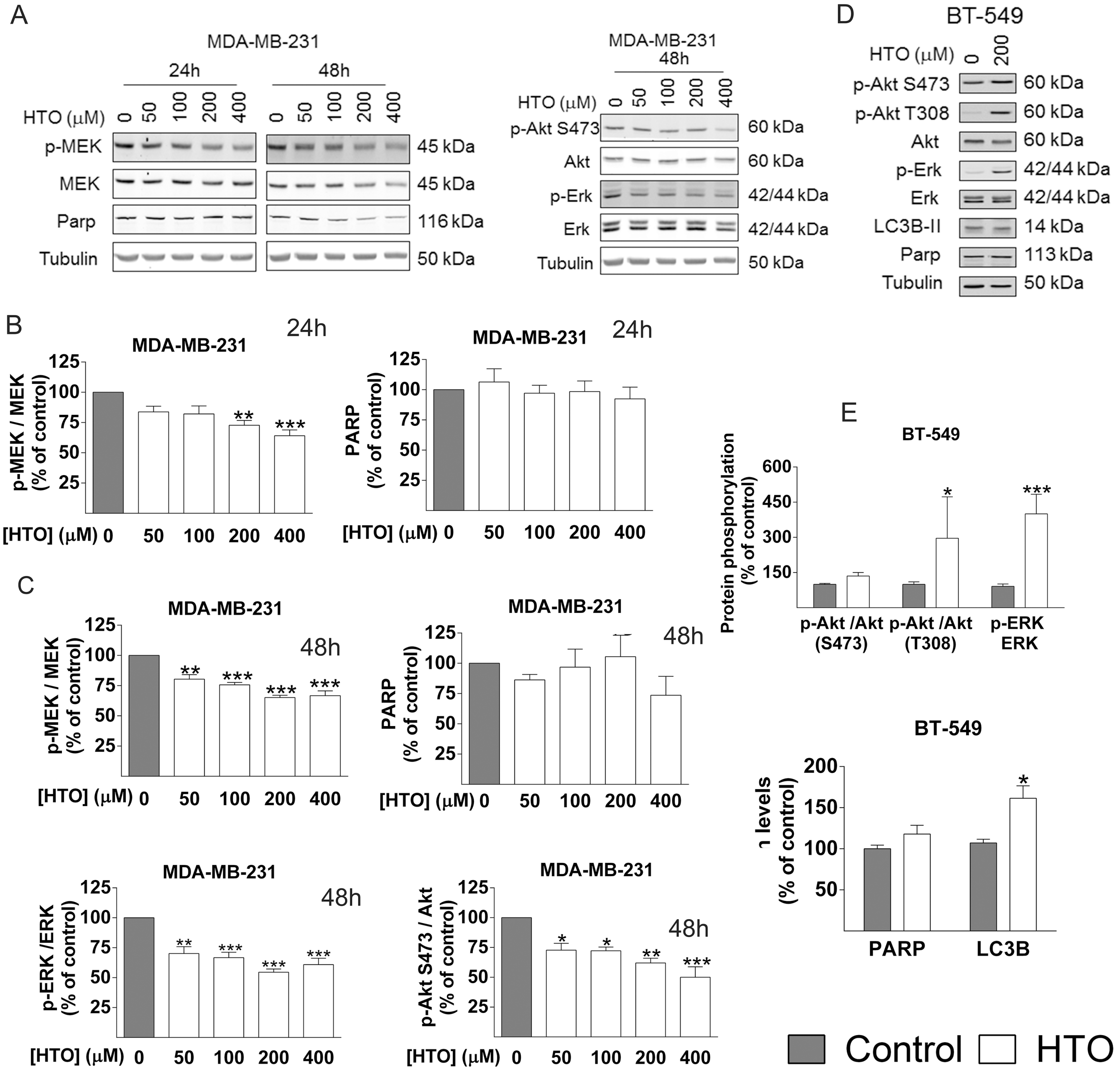

Supplementary Figure 3: Effect of HTO on phosphatases. MDA-MB-231 cells were serum starved, cultured for 24 h in the presence or absence of HTO (150 μM), for 2 h in the presence or absence of orthovanadate (100 nM) and stimulated for 15 min with EGF (100 ng/ml) or left untreated before the cell lysates were prepared for immunoblotting. Representative immunoblots are shown, the bars corresponding to the mean ± SEM values of 3 independent experiments: $^{*/+}p < 0.05$, $^{**}p < 0.01$, $^{***/+++}p < 0.001$ (ANOVA followed by Bonferroni's test).

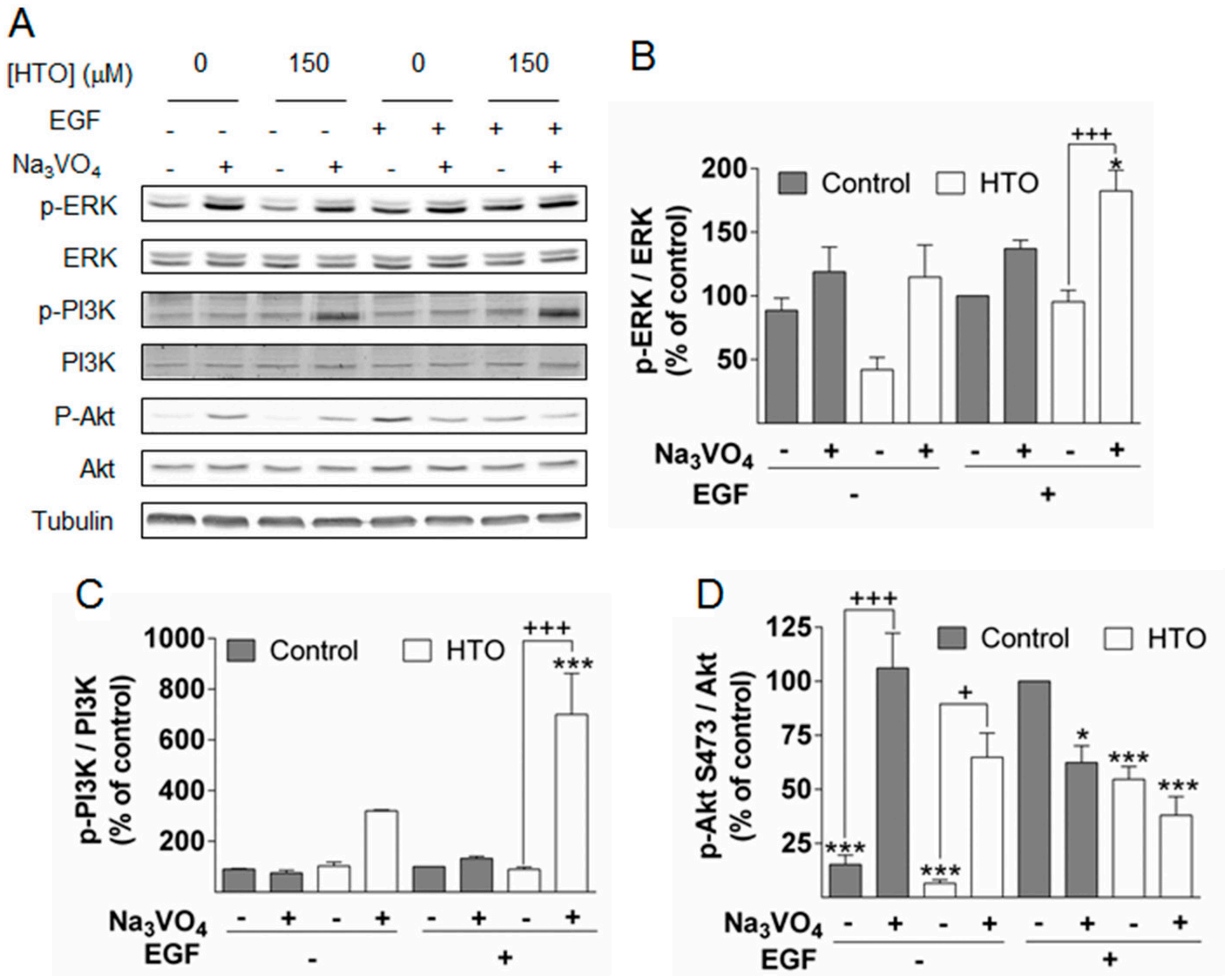

Supplementary Figure 4: Linear correlation for the 2OHOA mass and GC peak area. The concentrations of 2OHOA indicated were submitted transmethylation and half of the sample was then derivatized. The fatty acid levels were measured by GC, with 1 μl of each solution applied to the GC device.
